# PMEPA1 modulates YAP1 nuclear translocation to disrupt EMT subtypes and promote metastasis in Biliary tract cancer

**DOI:** 10.1038/s41419-026-08684-3

**Published:** 2026-04-03

**Authors:** Wenwen Xu, Chaoqun Ma, Pin Li, Xinxing Lyu, Ruitang Xu, Kaiwen Sheng, Ting Zhang, Haoqiang Sun, ZhaoXiang Zhang, Ziyi Wang, Hongguang Li, Dianhao Guo, Shuhong Huang

**Affiliations:** 1 Department of Hepatobiliary Surgery, Shandong Provincial Hospital Affiliated to Shandong First Medical University, Jinan, Shandong China; 2 School of Clinical and Basic Medical Sciences, Shandong First Medical University & Shandong Academy of Medical Sciences, Jinan, Shandong China; 3 College of Life Sciences, Shandong Normal University, Jinan, Shandong China; 4 School of Basic Medical Sciences, Cheeloo College of Medicine, Shandong University, Jinan, Shandong China

**Keywords:** Tumour biomarkers, Transcriptomics

## Abstract

Biliary tract cancer (BTC) is an aggressive tumor with poor prognosis and limited treatment options. Epithelial-mesenchymal transition (EMT) significantly contributes to BTC metastasis, yet the critical regulators and underlying molecular mechanisms of EMT remain poorly unclear. In our study, we conducted an integrative analysis of single-cell RNA sequencing (scRNA-seq) data from 47 BTC samples, identifying two EMT-enriched epithelial subpopulations and a BTC specific EMT gene set. Using the EMT gene set, we classified BTC patients and noticed that those with elevated EMT scores had worse prognoses. Additionally, multi-omics analyses identified PMEPA1 as a pivotal EMT regulator, with its high expression levels correlating with adverse prognosis and distant metastasis. Functional assays revealed that PMEPA1 knockdown inhibits, while its overexpression promotes EMT progression by modulating Hippo-YAP signaling, specifically YAP1 nuclear localization. Moreover, we forecast and validated topoisomerase inhibitor SN-38 as a potential EMT-targeting agent. SN-38 effectively inhibited BTC cell migration and metastasis in vivo, likely by attenuating the transcriptional activity of PMEPA1 and its upstream regulator FOS. Overall, this research elucidates EMT-related molecular features in BTC, reveals PMEPA1 as a critical EMT driver via the Hippo-YAP1 pathway, and proposes SN-38 as a promising therapeutic candidate, offering new insights into BTC metastasis and precision therapy.

**Figure:**
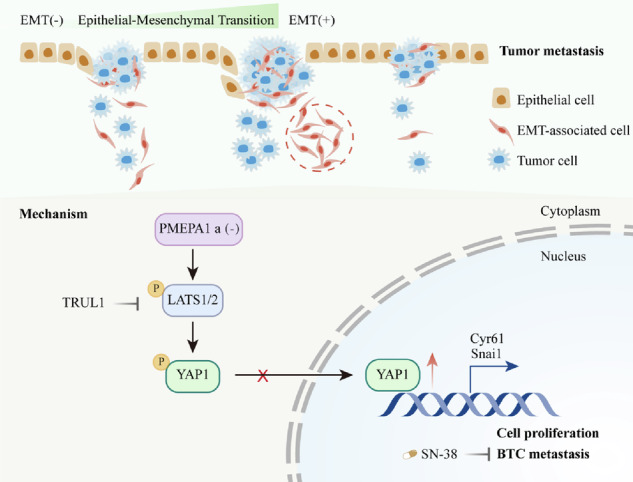
Our study identifies PMEPA1 as a central regulator of EMT and metastasis in BTC. The PMEPA1-a isoform bypasses the tumor-suppressive Hippo pathway by inhibiting LATS1/2 kinase activity. This liberates YAP1 to translocate into the nucleus and initiate a pro-EMT gene expression program. Finally, we demonstrate that the drug candidate SN-38 suppresses metastasis by interfering with this PMEPA1/YAP1 signaling module.

Our study identifies PMEPA1 as a central regulator of EMT and metastasis in BTC. The PMEPA1-a isoform bypasses the tumor-suppressive Hippo pathway by inhibiting LATS1/2 kinase activity. This liberates YAP1 to translocate into the nucleus and initiate a pro-EMT gene expression program. Finally, we demonstrate that the drug candidate SN-38 suppresses metastasis by interfering with this PMEPA1/YAP1 signaling module.

## Introduction

Biliary tract cancer (BTC) is an aggressive malignancy that arises from the epithelial cells of the biliary system. Over the past few decades, its incidence has been increasing worldwide, with the highest rates reported in Asian countries [1]. BTC includes several subtypes: intrahepatic cholangiocarcinoma (iCCA), perihilar cholangiocarcinoma (pCCA), distal cholangiocarcinoma (dCCA), and gallbladder cancer (GBC), with most cases classified as adenocarcinomas [2]. BTC is marked by its aggressiveness, high mortality rate, short survival duration, with a less than 20% 5-year survival rate [3, 4]. Thus, understanding the molecular mechanisms driving BTC invasiveness is essential for identifying novel therapeutic targets, improving treatment effectiveness, and enhancing patient outcomes.

Epithelial-mesenchymal transition (EMT) is recognized as a key mechanism driving BTC metastasis [5, 6]. EMT is orchestrated by numerous core EMT transcription factors (TFs), including snail family transcriptional repressors 1/2 (SNAI1/2), twist family bHLH transcription factors 1/2 (TWIST1/2), and zinc finger E-box binding homeobox factors 1/2 (ZEB1/2). These TFs promote EMT by suppressing epithelial genes like CDH1 (E-cadherin) and TJP1 (ZO-1), which reduces cell adhesion. Simultaneously, they induce the expression of mesenchymal genes including VIM (vimentin) and CDH2 (N-cadherin), facilitating cell migration and contributing to cancer cell dissemination and drug resistance [7]. In BTC, EMT is initiated by aberrant epithelial-origin primary cancer lesions, causing a shift from epithelial to mesenchymal phenotypes [8, 9]. Multiple signaling pathways regulate EMT, including TGFB, Wnt, Notch, and Hippo/YAP pathways. The interplay of various signaling pathways and TFs underscores EMT’s crucial role in tumor invasiveness, metastasis, and therapeutic resistance.

Through EMT, tumor cells acquire mesenchymal features, penetrate basement membranes, enter circulation systems (blood or lymphatic), and colonize distant organs, thereby enhancing metastasis [10, 11].

Recently, research has highlighted the significance of understanding EMT heterogeneity and subtype-specific molecular characteristics in cancer therapy. Studies in bladder and liver cancers have shown that different tumor subtypes exhibit distinct EMT characteristics and gene expression profiles, which correlate with varying levels of invasiveness and prognosis [12, 13]. The EMT score has been recognized as a valuable marker for predicting tumor metastasis [14]. For instance, in head and neck squamous cell carcinoma, EMT levels are associated with metastatic potential and adverse pathological features [15]. We previously identified EMT subtypes in esophageal squamous cell carcinoma by analyzing EMT molecular characteristics [16]. These discoveries underscore the pivotal role of EMT in cancer progression and emphasize the need to study its heterogeneity and subtype characteristics in BTC. A comprehensive understanding of EMT-related molecules and their variability in BTC is crucial for discovering new pathways to inhibit metastasis. This knowledge is also vital for the development of effective targeted therapies, ultimately leading to improved clinical outcomes. However, in BTC, there is limited reporting on the molecular heterogeneity and subtype-specific features of EMT.

Prostate transmembrane protein androgen-induced 1 (PMEPA1), located on chromosome 20q13, was first identified as an androgen-induced gene in prostate cancer. Recent pan-cancer studies have shown that PMEPA1 is overexpressed in several cancers, including bladder, prostate, and breast cancer. This overexpression suggests that PMEPA1 may serve as a potential prognostic marker with various clinical correlations [17, 18]. The biological functions of PMEPA1 exhibit substantial heterogeneity across different cancers, indicative of cancer-specific regulatory mechanisms [19].

PMEPA1 also modulates several signaling pathways and significantly impacts tumor metastasis by accelerating EMT. Research has demonstrated that PMEPA1 regulates reactive oxygen species (ROS) and insulin receptor substrate 1 (IRS1) signaling, and induces EMT to facilitate lung cancer metastasis [20]. In triple-negative breast cancer, PMEPA1 downregulates PHLPP1 activity and promotes EMT, exacerbating tumor progression [21, 22]. Additionally, research demonstrates that PMEPA1 promotes glioma progression by ubiquitination mediated degradation of LAST1 kinase, consequently suppressing Hippo signaling pathway activity [23]. These findings underscore PMEPA1’s crucial role in tumor invasiveness and metastatic potential. In terms of drug therapy, targeting PMEPA1 and its associated signaling pathways may enhance sensitivity to chemotherapeutic agents such as gemcitabine and cisplatin, potentially improving pancreatic cancer treatment efficacy [24]. However, the expression patterns, predominant isoforms, and biological roles of PMEPA1 in BTC remain largely unexplored.

Single-cell RNA sequencing (scRNA-seq) has become an vital method for studying complex tumors, such as BTC, due to its capability to analyze cellular heterogeneity and dynamic changes at the single-cell level [25]. This technology has revealed key molecular characteristics, including cell subpopulation composition, copy number variations, immune suppression and evasion mechanisms, tumor microenvironment, and responses to and resistance against neoadjuvant chemoradiotherapy [26]. We previously employed scRNA-seq to investigate the heterogeneous landscape and tumor microenvironment of dCCA, uncovering the complexity of tumor heterogeneity in dCCA [27]. Additionally, scRNA-seq has been used to explore EMT markers and processes, demonstrating considerable potential for BTC treatment and biomarker discovery. During EMT studies, scRNA-seq meticulously captures cell state transitions and intertumoral heterogeneity, addressing the limitations of earlier techniques that struggled to track single-cell EMT dynamics. Pastushenko et al. identified multiple cell states during EMT through single-cell sequencing, underscoring the complexity of EMT heterogeneity in tumors beyond a simplistic binary classification of epithelial or mesenchymal states [28].

This high-resolution analysis of cell states not only enhances our understanding of BTC mechanisms but also helps identify more precise therapeutic targets. Currently, the standard chemotherapy regimen for advanced BTC predominantly involves combining gemcitabine with other drugs. While this combination therapy has improved overall survival rates, its efficacy remains limited, with minimal long-term survival benefits for most patients [29, 30]. However, scRNA-seq enables a detailed examination of tumor heterogeneity, allowing us to optimize cancer treatment strategies. For example, single-cell analysis of cholangiocarcinoma has shown that transitions in CD8 + T cell states and Macro CD5L + -induced exhaustion are crucial in determining the response to combination therapy in intrahepatic cholangiocarcinoma patients. This insight opens potential avenues to counteract treatment resistance [31].

In this study, we analyzed single-cell RNA sequencing (scRNA-seq) data from 47 biliary tract cancer (BTC) samples. Through a combination of analytical approaches—including cell subpopulation identification, pseudotime analysis, and cNMF—we characterized the diversity and heterogeneity of cells within the BTC tumor ecosystem, constructing a comprehensive single-cell landscape of BTC. Our analysis revealed distinct epithelial–mesenchymal transition (EMT) cell clusters and identified a core set of 51 EMT-associated genes. Notably, we discovered a novel EMT marker in BTC, PMEPA1, which facilitates EMT by engaging with the Hippo–YAP signaling pathway, thereby enhancing the invasive and metastatic capacity of BTC cells. Furthermore, we classified BTC samples according to EMT subtypes and leveraged drug databases to identify potential EMT-targeted therapies for BTC. Mechanistically, we demonstrated that the EMT-potential drug SN-38 suppresses tumor metastasis by disrupting the binding of the transcription factor FOS to the PMEPA1 promoter region. Collectively, our findings underscore the therapeutic promise of targeting EMT-related genes to suppress BTC metastasis.

## Methods

### Patient samples

In this study, we collected and sequenced samples from three patients with hilar cholangiocarcinoma. Supplementary Table S1 includes comprehensive clinical information on these patients. None of the patients had undergone chemotherapy, radiotherapy, or drug treatment before tumor resection. During surgical resection, we obtained fresh paired tumor and non-tumor hilar bile duct tissues. All adjacent non-malignant hilar bile duct tissues were collected from at least 5 cm away from the tumor. The six clinical specimens were sourced from Shandong Provincial Hospital, affiliated with Shandong First Medical University. This study adhered to the ethical standards set by the Shandong First Medical University Medical Ethics Committee, and we obtained informed consent from all patients. Each patient provided written informed consent for both sample collection and data analysis.

### Preparation of single-cell suspension

Fresh tissues were rinsed in PBS, minced into 1–2 mm³ pieces, and digested with trypsin at 37 °C for 30 min with gentle pipetting. Digestion was stopped with FBS-containing DMEM, and the suspension was filtered through 70 μm and 40 μm strainers. Cells were centrifuged (500 × g, 5 min, 4 °C), resuspended in PBS, and counted using a Luna fluorescence cell counter. Only samples with >80% viability, < 5% debris, and < 5% cell clumping were used. The final cell concentration was adjusted to 700–1200 cells/μL for single-cell RNA-seq.

### Single-cell RNA-seq library

Single-cell capture, library preparation, and sequencing were performed using the 10X Genomics Chromium Single Cell 3’ Reagent Kit v3.1. Cell suspensions, gel beads, and oil were loaded into Chromium Chip G to generate GEMs containing individual cells and barcoded beads. Inside GEMs, cells were lysed, and mRNA was reversed transcribed into barcoded cDNA tagged with UMIs. After emulsion breakage, cDNA was PCR-amplified, fragmented (200–300 bp), end-repaired, A-tailed, and ligated with sequencing adapters (P5/P7 with dual indices). Library concentration and size were measured using a Qubit 3.0 fluorometer and Agilent 2100, respectively. Final libraries were quantified by qPCR and sequenced on an Illumina platform (paired end). Raw data were quality-checked with FastQC and processed by Cell Ranger (10X Genomics), aligned to the GRCh38 reference genome. Barcodes and UMIs were used for quantification to generate the gene expression matrix. Sequencing was performed by Annoroad Gene Technology Co., Ltd. (Beijing, China).

### scRNA-seq data analysis

All single-cell datasets were merged into a single Seurat object using the merge function (Seurat v4.3.0) and filtered based on sequencing quality metrics. [32] Samples with fewer than 1000 cells were removed. Low-quality cells were excluded based on the following criteria: fewer than 500 or more than 6000 detected genes, > 25% mitochondrial gene content, or >0.1% hemoglobin gene expression. Genes not detected in at least three cells were also discarded. Potential doublets were identified and removed using the DoubletFinder R package (v2.0.3) [33]. The remaining cells were considered high-quality and normalized using NormalizeData to obtain a standardized barcode count matrix. Highly variable genes were identified using FindVariableFeatures, and the top 2000 genes were selected. ScaleData was used to regress out UMI counts and mitochondrial content. PCA was performed via RunPCA, and Harmony was used to correct for batch effects. Dimensionality reduction and clustering were conducted using RunUMAP, FindNeighbors, and FindClusters.

We determined cell types through the expression of established marker genes. T cells were identified by CD3D, CD3E, and CD7, B cells by CD79A, MS4A1, IGKC, and MZB1, dendritic cells (DC) by CLEC10A, CD1C, and FCER1A, epithelial cells by EPCAM, KRT19, and KRT8, cycling cells by UBE2C, CDK1, and MKI67, macrophages by MRC1, CD163, and CD14, monocytes by S100A8, S100A9, and FCN1, fibroblasts by ACTA2, COL3A1, and COL1A1, endothelial cells by PECAM1, VWF, and CD34, and mast cells by TPSAB1, TPSB2, and TPSD1.

### Gene set signature scores

We used the default parameter of the “AddModuleScore” function from Seurat to calculate the average expression levels of target gene set value at the single-cell level [34].

### Signaling pathway enrichment analysis

To investigate pathway enrichment variation among different subtypes, we employed Gene Set Variation Analysis (GSVA, version 1.48.3). We focused on 50 hallmark pathways sourced from the Molecular Signatures Database (MSigDB, available at https://www.gsea-msigdb.org/gsea/msigdb).

### EMT expression programs

we utilized Consensus Non-negative Matrix Factorization (cNMF) (https://github.com/dylkot/cNMF) to infer gene expression programs for each sample matrix in BTC. During the cNMF process, we set 500 decomposition iterations to calculate the consensus matrix, assessing the robustness and consistency of gene modules. The optimal value of k is determined to be 2. After selection, we identified 74 group-specific expression programs. We then subjected these 74 programs to clustering, identifying five modules. To investigate the importance of these modules in terms of their functionality, we utilized HALLMARK pathways to perform pathway enrichment analysis. For each program, we identified the top 50 genes and kept the 5 most enriched pathways. Particularly, genes associated with the “EPI EMT_MESENCHYMAL_ TRANSITION” pathway were clustered within the same module and identified as core EMT genes in BTC.

### Trajectory inference

We performed trajectory analysis using the R package Monocle (version 2.22.0) to examine the dynamic EMT process among epithelial cell clusters [35]. We constructed the statistical model by employing the “estimateSizeFactors” and “estimateDispersions” functions. Dimensionality reduction was achieved using the “reduceDimension” function, and the “orderCells” function was employed to arrange cells along the pseudotime trajectory. To infer the EMT cohort, we selected common up-regulated and down-regulated genes as ordering genes (see Supplementary Table S4). Additionally, we combined trajectory analysis with Branch Expression Analysis Modeling (BEAM) to pinpoint genes exhibiting branch-dependent expression.

### TCGA and RNA-seq gene expresion analysis

We collected RNA-seq data for cholangiocarcinoma tissues from The Cancer Genome Atlas (TCGA) via the UCSC Xena database https://xenabrowser.net/datapages/. This dataset included 9 normal tissue samples and 36 BTC samples. Additional data from GSE107943 were retrieved from the GEO database https://www.ncbi.nlm.nih.gov/geo/, with accession numbers provided in Supplementary Table S2. We conducted differential expression analysis using DESeq2 [36] to identify differentially expressed genes (DEGs). Following the filtering of DEGs, we identified genes that were significantly differentially expressed, defined by a *p*-value < 0.05 and an absolute log2 fold change > 1.

### KEGG, GO, and Wikipathway analysis

We conducted Kyoto Encyclopedia of Genes and Genomes (KEGG) pathway, Gene Ontology (GO) enrichment and Wikipathway analyses using the Database for Annotation, Visualization, and Integrated Discovery (DAVID, https://david.ncifcrf.gov/ [37].

### Unsupervised consensus clustering

We performed unsupervised hierarchical clustering using the ConsensusClusterPlus package (version 1.66.0) [38] in R to categorize epithelial cells into EMT-high and EMT-low groups. This categorization was derived from 51 genes identified through cNMF. We set the analysis parameters as follows: repetitions = 500, with all other parameters at their default settings.

### Survival analysis

We obtained clinical data from TCGA and conducted Kaplan-Meier survival analysis on 36 tumor samples, excluding the 9 normal samples. We utilized the R packages survival and survminer and employed the log-rank test to evaluate statistical significance for this analysis.

### Prediction of drug sensitivity

We applied the oncoPredict R package (version 1.2) [39] to assess the predictive value of genes differentially expressed in epithelial cell subclusters regarding drug sensitivity. For this research, we used gene expression profiles from GDSC1 and GDSC2 https://www.cancerrxgene.org/, which served as the training set. We employed the “calcPhenotype” function to predict the half-maximal inhibitory concentration (IC50) for all drugs in BTC samples. We then applied a methodology incorporating with low Log2 Fold Change and high average sensitivity scores to predict candidate drugs for targeting EMT in BTC.

### SCENIC analysis

Gene regulatory networks were reconstructed using pySCENIC (version 1.3.1, https://github.com/aertslab/pySCENIC). First, co-expression modules were inferred using GRNBoost2 based on our processed scRNA data. Second, direct transcriptional factors (TF)-to-target interactions were identified through DNA motif analysis. Finally, regulon activity was scored for each cell using AUCell. All analyses were performed using default parameters.

### Cell culture

We cultured cholangiocarcinoma cell lines HUCCT1 (FuHeng BioLogy, FH0572) and RBE (OriCell, H1-1801), including shNC, shPMEPA1 and over expression variants, in RPMI-1640 medium supplemented with 10% fetal bovine serum (Gibco, A3160802), 100 U/mL penicillin, and 100 µg/mL streptomycin (Scolarbio, P1400).

### Stable cell lines

We infected HUCCT1 and RBE cells with LV-PMEPA1-RNAi, LV-CON313 LV-PMEPA1-OE and LV-CON lentiviral plasmids (Gene Chem, China). The cells were then selected with 1 μg/mL puromycin (MedChemExpress, HY-K1057) for 2 weeks, successfully establishing PMEPA1 knockdown cell lines and their corresponding control cell lines in both HUCCT1 and RBE. The target sequences in Supplementary Table S6.

### Quantitative real-time PCR

Total RNA was extracted from the cells using the UltraPure RNA Kit (Sparkjade, AC0205), and mRNA was reverse transcribed into cDNA using the cDNA synthesis kit (CWBIO, CW2569M). The SYBR Green PCR master mix (CWBIO, CW0957) was utilized to carry out quantitative PCR. β-Actin served as the internal control, and mRNA expression levels were quantified using the 2^−ΔΔCt^ method. The reagents used are listed in Supplementary Table S5. The primer sequences in Supplementary Table S7.

### Western blotting

We extracted total protein from cells using RIPA buffer (CWBIO, CW2333S) combined with Protease Inhibitor Cocktail (MedChemExpress, HY-K011). We then separated the proteins by SDS-PAGE and transferred them onto a PVDF membrane. Following a 5% non-fat milk blocking step, we incubated the membrane overnight at 4 °C with primary antibodies. Subsequently, we incubated the membrane at room temperature for 1 hour with the corresponding secondary antibodies. We visualized the protein bands using the hypersensitive ECL chemiluminescence detection kit. Detailed information on the antibodies and reagents used is provided in Supplementary Table S5.

### Cell proliferation assays

We seeded shNC and shPMEPA1 BTC cells into 96-well plates at a density of 5000 cells per well and allowed them to attach and grow. At 0, 24, 48, and 72 h, we added 10 µL of CCK-8 reagent (MedChemExpress, HY-K0301) to each well and incubated for 1 hour. We then measured the absorbance at a wavelength of 450 nm.

### Colony formation

We seeded shNC and shPMEPA1 BTC cells into 6-well plates at a density of 200 cells per well and cultured them for 14 days, refreshing the medium every 3 days. After the incubation period, we fixed the resulting colonies with 4% paraformaldehyde for 20 min and then stained them with 0.1% crystal violet for 20 min.

### Subcutaneous Xenograft Model

HUCCT1 cells from the shNC and shPMEPA1 groups in the logarithmic growth phase were resuspended in PBS and mixed 1:1 with Matrigel to a final concentration of 8 × 10⁷ cells/mL. A total of 125 μL of the cell suspension was subcutaneously injected into the right axilla of 5-week-old male BALB/c nude mice using an insulin syringe. Each group consisted of six mice. Tumor length and width were measured, and tumor volume was calculated using the formula: volume (mm³) = ½ × (length × width²). Mice were sacrificed for 19 days post-inoculation, and tumors were photographed, harvested, and weighed. The animal experiments were conducted using a randomization method.

### Cell migration assay

Cells were resuspended in serum-free medium at 5 × 10⁵ cells/mL, and 200 μL was added to the upper chamber of a Transwell insert. The lower chamber contained 600 μL medium with 20% FBS. After 24 h incubation, cells were fixed with 4% paraformaldehyde for 20 min, washed with PBS, and stained with 0.1% crystal violet for 30 min. Non-migrated cells on the upper surface were removed, and migrated cells were imaged and counted under a microscope.

### Cell invasion assay

We used the transwell assay to evaluate cell invasion. Invasion assays employed basement membrane matrix (Corning, 356234) transwell inserts. The subsequent experimental procedures for the cells were conducted in the same method as the migration assay.

### Tail Vein Injection Metastasis Model

Stable GFP-expressing shNC and shPMEPA1 HUCCT1 cells in the logarithmic growth phase were resuspended in PBS and adjusted to a final concentration of 1 × 10⁶ cells/100 μL. Using an insulin syringe, 100 μL of the cell suspension was slowly injected into the tail vein of 5-week-old male BALB/c nude mice. Each group included at least five mice. After injections, mice were continuously monitored for general health status. Forty days later, mice were sacrificed, and lungs were harvested, photographed, and weighed. The lung tissues were then fixed in 4% paraformaldehyde for 24 h, followed by paraffin embedding and sectioning. Immunohistochemical staining was performed to assess the number and size of metastatic nodules.

Stable luciferase-expressing NC and PMEPA1-OE HUCCT1 cells were used to construct lung metastasis models following the aforementioned method. One week later, the mice were divided into the NC + DMSO, PMEPA1-OE + DMSO, NC + SN-38, and PMEPA1-OE + SN-38, with 5 mice in each group. Intraperitoneal injections of DMSO or SN-38 were administered every three days, with the injection volume calculated based on the weight of each mouse (5 mg/kg). On the 7th, 14th, and 21st days after the start of treatment, the lung metastatic was monitored using an in vivo imaging system. At the end of the experiment (on the 21st day), the mice were sacrificed and lung tissues were collected for subsequent analysis. The animal experiments were conducted using a randomization method.

### Immunohistochemistry assay

We deparaffinized and rehydrated paraffin-embedded tissue sections using alcohol. We performed antigen retrieval by microwave heating. We used normal goat serum (CWBIO, CW0130S) as a blocking agent. We stained the sections overnight at 4 °C with the primary antibody. For immunostaining, we used goat anti-rabbit/mouse HRP-labeled polymer as the secondary antibody and detected signal intensity under a microscope using DAB. We counterstained the nuclei with hematoxylin, differentiated, blued, and then dehydrated the sections with gradient alcohol. Finally, we mounted the sections with neutral gum and photographed them. Detailed information about the reagents used is provided in Supplementary Table S5.

### Drug Treatment

The shNC and shPMEPA1 groups of cells were treated with 10 μM TRULI (MedChemExpress, HY-138489) for 24 h. HUCCT1 or RBE cell were treated with 0.05 μM docetaxel (MedChemExpress, HY-B0011), 1 μM epothilone B (MedChemExpress, HY-17029), 0.5 μM elesclomol (MedChemExpress, HY-12040), 0.2 μM gemcitabine (MedChemExpress, HY-17026), 50 μM oxaliplatin (MedChemExpress, HY-17371), 40 μM SN-38 (MedChemExpress, HY-13704), 0.06 μM vinorelbine ditartrate (MedChemExpress, HY-12053A) for 24 h. Detailed information about the reagents used is provided in Supplementary Table S8.

### Immunofluorescence Staining

Cells were seeded at 5 × 10⁵ cells/mL in 24-well plates containing coverslips and incubated for 24 h. After PBS washes, cells were fixed with 4% paraformaldehyde for 30 min, washed again, and blocked with normal goat serum. Coverslips were incubated overnight at 4 °C with primary antibodies, followed by incubation with CoraLite 488-conjugated anti-rabbit or CoraLite 594-conjugated anti-mouse secondary antibodies. Nuclei were counterstained with DAPI. Antibody details are listed in Supplementary Table S5.

### Cytoplasmic and Nuclear Protein Extraction

Cytoplasmic and nuclear protein fractions were isolated using the Minute™ Cytoplasmic and Nuclear Extraction Kit (Invent Biotechnologies, USA) according to the manufacturer’s instructions. The extracted protein components were subjected to ultrasonic disruption to obtain high-quality proteins. GAPDH was used as a cytoplasmic loading control, and Lamin B1 was used as a nuclear loading control, as recommended by the kit protocol.

### Prediction of TFBS and Plasmid Construction

Putative transcription factor binding sites (TFBS) on the target gene promoter were predicted using the SCENIC, hTFtarget (https://guolab.wchscu.cn/hTFtarget/), and JASPAR databases (https://jaspar.elixir.no/). Based on the consensus sites, both wild-type and mutant (carrying site-specific mutations in the predicted TFBS) promoter reporter plasmids were constructed via a homologous recombination kit.

### Dual-luciferase reporter assay

We cloned the promoter region of the PMEPA1 gene into the pGL3-Basic plasmid to obtain the luciferase reporter gene pGL3-Basic-PMEPA1-WT. On this basis, the original sequence (CGCTGACGTCAGAC) was mutated by seamless cloning site-directed mutagenesis technology to (CGCGACCGGACGAC), and the resulting plasmid was named pGL3-Basic-PMEPA1-MUT. After plasmid construction was completed, the HUCCT1 and RBE cells were seeded and transfected with the plasmid the next day. After 24 h, SN-38 drug was added, and the drug treatment was carried out for 24 h. Then, we used the Dual-Luciferase Reporter Gene Assay Kit (Beyotime, RG027) to measure the luciferase activity in the cells according to the manufacturer’s instructions.

### Statistical analysis

All data were presented as the mean ± standard deviation (SD) and analyzed using GraphPad Prism 9. We performed statistical analysis using the two-sided t-test for comparisons between two groups and one-way ANOVA with Bonferroni correction for comparisons among multiple groups. For CCK-8 assays and fluorescence imaging of mice, we used two-way ANOVA with Bonferroni correction. The cell experiments and in vitro experiments included three biological replicates. The number of nude mouse tumor samples was 6. The number of nude mouse tail vein lung metastasis model samples was 5. We considered a *p*-value less than 0.05 to be statistically significant. All data were independent of the samples, with homogenous variances, and conformed to a normal distribution.

## Results

### Single-cell transcriptional landscape of biliary tract cancer

**Fig. 1:**
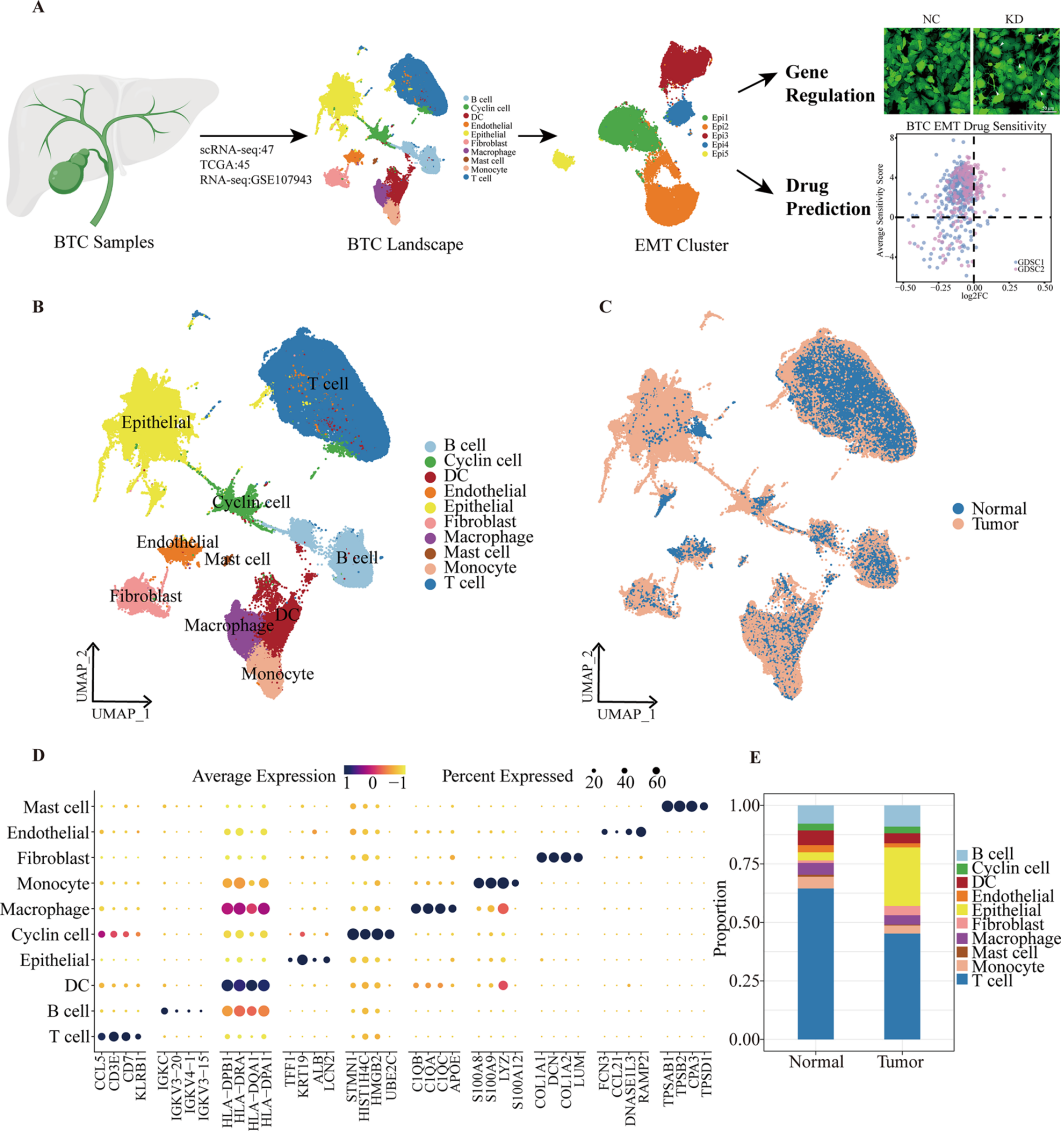
Single-cell transcriptional landscape of biliary tract cancer. **A** Schematic of the data analysis workflow for the BTC single-cell transcriptome. The scale bar represents 50 μm. **B**, **C** UMAP plots showing the 159,713 high quality single cells from 47 samples in BTC, colored by cell type (**B**) and tissue (**C**). **D** Dot plot illustrates the top 4 marker gene expression levels in various cell subtypes. The percentage of cells expressing the marker gene is represented by dot size, while dot color denotes the average expression level. **E** Bar plot showing the proportion of the 10 cell types in Normal and Tumor tissues.

To develop a high-quality single-cell atlas of BTC, we conducted 10X single-cell sequencing on postoperative tumor and normal tissues from three patients and integrated 41 scRNA-seq samples from the GEO database. This integration resulted in a total of 47 scRNA-seq datasets, including 33 tumor tissues and 14 normal tissues (Supplementary Table 1). Our research workflow includes BTC sample collection, construction of the BTC landscape, identification of EMT clusters, and subsequent analysis of gene regulation and drug predictions. This comprehensive approach allows us to analyze the EMT phenomenon in BTC thoroughly and predict potential EMT-targeted drug therapies (Fig. 1A).

Following quality control, integrative analysis, and marker gene annotation, we generated a high-resolution BTC single-cell atlas encompassing 159,713 cells (Figure S1A). This atlas comprised 35 cell clusters and identified 10 cell types (Fig. 1B, S1B): B cells (*N* = 13,983), cycling cells (*N* = 4571), DC (*N* = 7700), endothelial cells (*N* = 3348), epithelial cells (*N* = 30,745), fibroblasts (*N* = 5086), macrophages (*N* = 6895), mast cells (*N* = 768), monocytes (*N* = 6195), and T cells (*N* = 80,422).

The UMAP visualization illustrates notable differences in the distribution of normal and tumor cells (Fig. 1C). We confirmed the reliability of the cell clusters in the single-cell atlas using established marker genes (Fig. 1D). A comparative analysis of cell proportions between tumor and normal tissues revealed variations in the distribution of each cell type. Specifically, the proportion of tumor epithelial cells was significantly higher than that in non-cancerous tissues, showing the most pronounced difference (Fig. 1E, S1C). These findings underscore the considerable tumor heterogeneity in BTC and suggest that epithelial cells may play a crucial role in the progression of biliary tract cancer.

### Transcriptomic heterogeneity of Epithelial cells in BTC

**Fig. 2:**
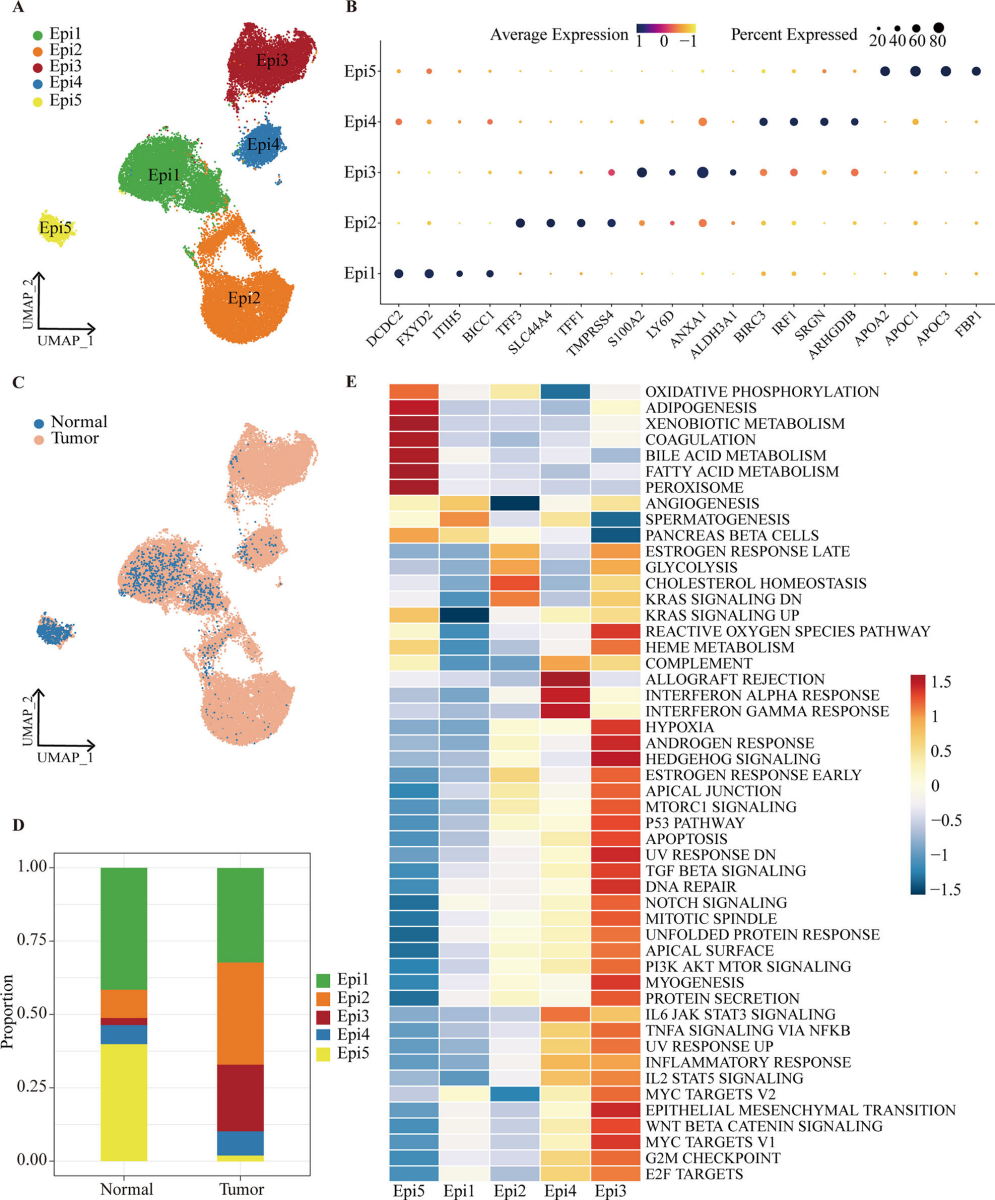
Transcriptomic heterogeneity of Epithelial cells in BTC. **A** UMAP plot of Epithelial cell subclusters. The 5 distinct subclusters are colored differently. Each dot denotes a single cell. **B** Dot plot of the expression levels of the marker genes for each cell subtype. The percentage of cells expressing the marker gene is represented by dot size, while dot color denotes the average expression level. **C** UMAP plot of Normal and Tumor cells by their Epithelial cell type. **D** Bar plot showing the proportion of distinct Epithelial cell subclusters in Normal and Tumor tissues. **E** Heatmap illustrating variations in hallmark pathway activation (scored by GSVA) in 5 Epithelial cell subclusters.

To analyze the transcriptional heterogeneity of epithelial cell subclusters in BTC, we re-clustered epithelial cells into five distinct subclusters, labeled Epi 1 through Epi 5 (Fig. 2A, S2A). We validated the characteristic gene expression profiles of these subclusters by analyzing marker gene expression (Fig. 2B, S2B). Specifically, the Epi1 subcluster shows high expression of tumor proliferation genes such as DCDC2, FXYD6, ITIH5, and BICC1. The Epi2 subcluster is distinguished by genes involved in regulating tumor energy metabolism, including TFF3, SLC44A4, TFF1, and TMPRSS4. The Epi3 subcluster exhibits elevated levels of genes related to tumor invasion, such as S100A2, LY6D, ANXA1, and ALDH3A1. The Epi4 subcluster is characterized by high expression of inflammation-related genes, including BIRC3, IRF1, SRGN, and ARHGDIB. Lastly, the Epi5 subcluster is marked by high expression of lipid metabolism genes, such as APOA2, APOC1, APOC3, and FBP1.

We compared the distribution of epithelial cell subclusters between tumor and normal tissues and observed significant differences. Subclusters Epi1, Epi2, Epi3, and Epi4 were predominantly found in tumor tissues, while Epi5 was almost exclusively present in normal tissues (Fig. 2C). Further proportional analysis indicated that Epi2, Epi3, and Epi4 subclusters were notably amplified in tumor tissues (Fig. 2D). To comprehend the functional properties of these subclusters of epithelial cells more thoroughly, we performed GSVA to examine their activity across various biological pathways. The results indicate that Epi1 is closely associated with tumor proliferation, specifically through the Myc targets V2 pathway. Epi2 is predominantly involved in cholesterol homeostasis and glycolysis, highlighting its crucial role in regulating tumor energy metabolism. Epi3 shows significant activity in EMT-related pathways, including TGFB, Wnt, and Notch signaling, suggesting its pivotal role in the EMT process and its contribution to tumor cell invasion and metastasis. Epi4 appears to be critical in regulating the tumor microenvironment, particularly through inflammatory responses. Epi5 plays a vital role in maintaining cellular metabolic balance and normal function, primarily through oxidative phosphorylation (Fig. 2E). These findings reveal differences in distribution and functional pathways among epithelial cell subclusters in both tumor and normal tissues, highlighting their potential roles in BTC progression. The marked increase in Epi3 and Epi4 in tumors and their involvement in EMT-related pathways suggest that these cells may be integral to BTC invasion and metastasis. This provides new insights into the molecular mechanisms underlying BTC and suggests potentially targeted therapeutic strategies.

### Epi3 and Epi4 are the key epithelial cell subsets of BTC EMT

**Fig. 3:**
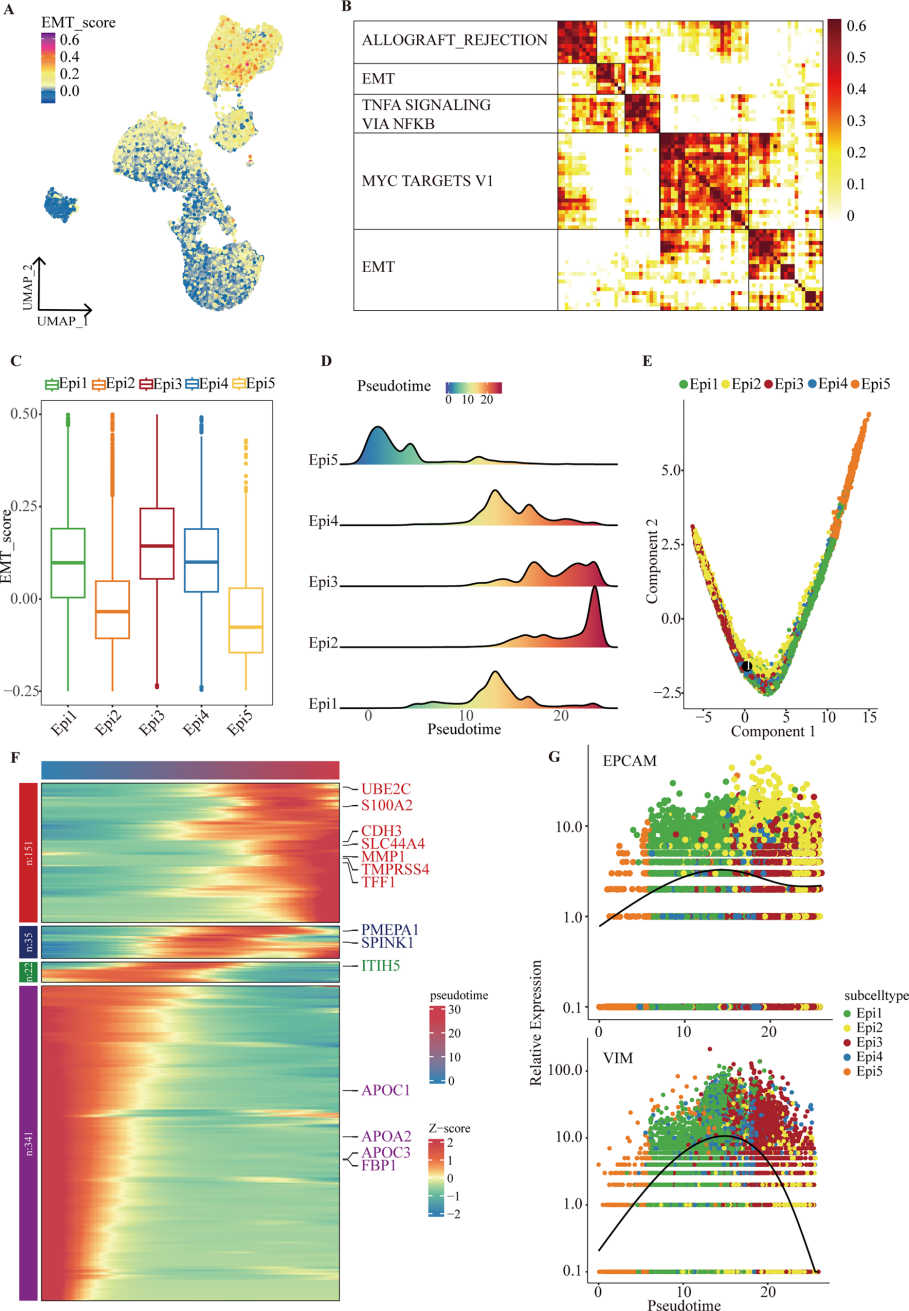
Epi3 and Epi4 are the key epithelial cell subsets of BTC EMT. **A** UMAP plot showing EMT score (scored per cell by hallmark EMT gene sets) in 5 Epithelial cell subclusters. **B** Heatmap illustrates the pairwise correlations among 5 modules derived from 74 expression programs, with annotated on the left section are the pertinent hallmark pathways. **C** Box plots depicting the signature scores of 51 EMT core genes, colored by its epithelial cell subtype. The boxes represent the lower quartile, middle quartile, and upper quartile, while the whiskers extend to 1.5 times the interquartile range (IQR). **D** Cell distribution of each epithelial cell subtype along with pseudotime. **E** Progression of EMT among epithelial cell subtypes. **F** Heatmap illustrates the pseudotime and gene dynamics of the EMT process. The differentially expressed genes are clustered hierarchically into four groups. Each gene group is shown in the right panel. **G** Temporal variation in EPCAM and VIM. Each point represents an individual cell, with colors indicating the cell subtypes.

To determine the EMT-related clusters of epithelial cells, we first performed an EMT scoring analysis of the epithelial cell subtypes using the Hallmarker EMT gene set. The analysis revealed that Epi3 and Epi4 had the highest EMT scores (Fig. 3A), which aligns with our previous results (Fig. 2E). This finding suggests that these two subclusters may play crucial roles in the EMT transition.

Given the variability in EMT marker expression across different tumors and the lack of well-defined EMT markers in BTC, we employed cNMF to further investigate specific EMT-related molecules in BTC. We conducted a detailed functional analysis of all epithelial cell subclusters based on their sample origin, resulting in the identification of 74 functional programs, each defined by the top 50 genes. These programs were then grouped into five distinct modules. Notably, the second and fifth functional modules were associated with EMT, underscoring their core functions in the EMT process (Fig. 3B). We compiled the genes from these modules into a BTC-specific EMT gene list, which includes 51 genes (Supplementary Table 3). Additionally, we calculated the EMT scores for various epithelial cell subclusters using the BTC-specific EMT gene list. Notably, the Epi3 and Epi4 subclusters consistently exhibited the highest EMT scores, further confirming their central roles in EMT (Fig. 3C, S3A).

We also conducted pseudotime trajectory analysis to model the state transitions of epithelial cell clusters throughout the EMT process. The analysis revealed a transition trajectory from Epi5, Epi1, and Epi4 to Epi2 and Epi3, clearly illustrating the dynamic progression of EMT (Fig. 3D–F, S3B). During this transition, the expression of the epithelial marker EPCAM decreased progressively from Epi1 and Epi4 to Epi3. Conversely, the expression levels of the EMT markers VIM and CDH2 increased progressively from Epi1 and Epi4 to Epi3 (Fig. 3G, S3C, S3D). This shift in marker expression further validates the dynamic changes occurring in cell subclusters during EMT. Consequently, it is inferred that Epi3 and Epi4 are key cell populations undergoing continuous EMT.

### Differential genes and heterogeneity in epithelial cells

**Fig. 4:**
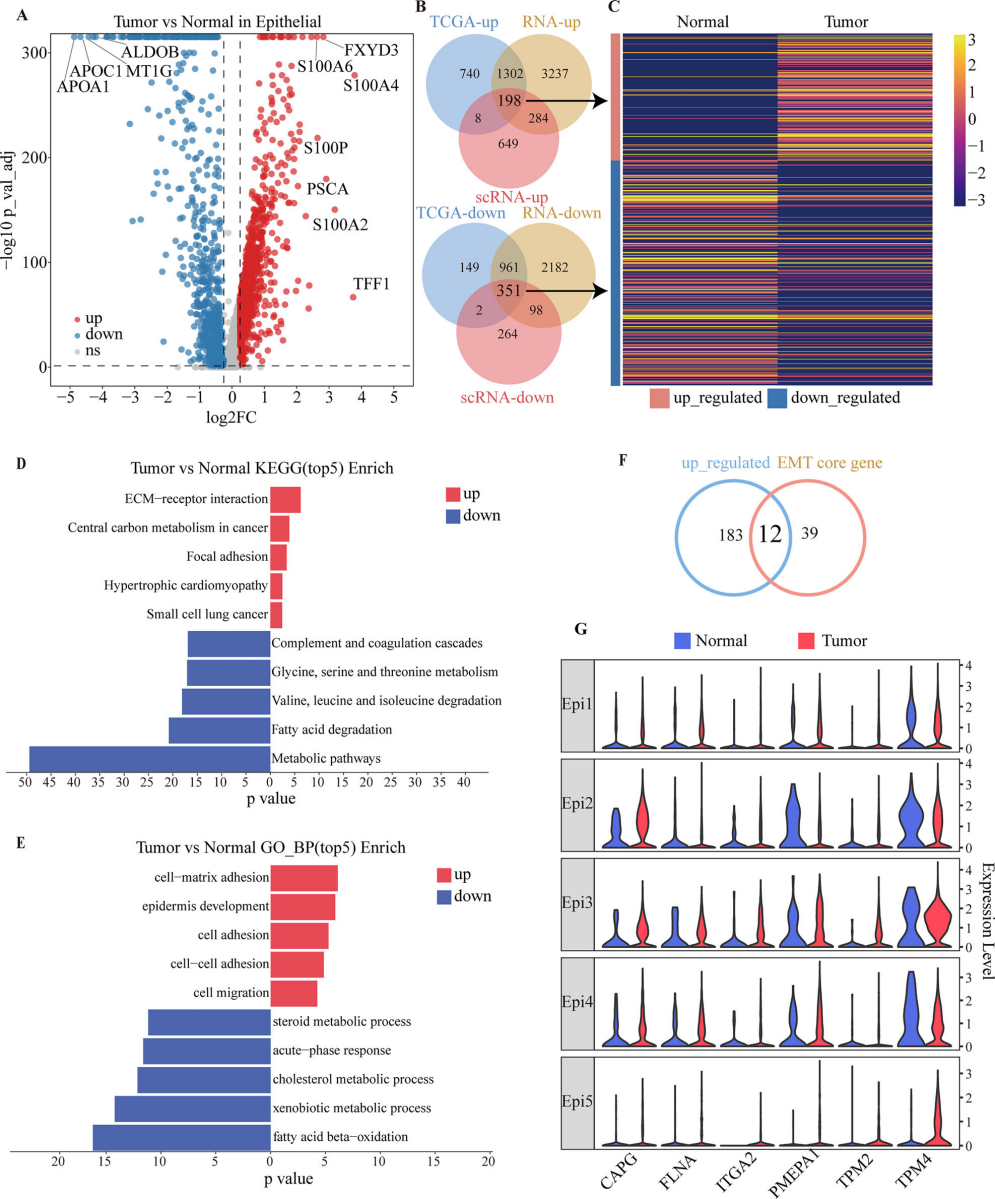
Differential genes and heterogeneity in epithelial cells. **A** Volcano plots displaying the DEGs between Normal and Tumor epithelial cells. Each dot denotes one gene. Genes with Log2FoldChange < −0.25 are down_regulated, those with Log2FoldChange > 0.25 are up_regulated, and gray dots indicate non-significant genes. **B** Venn diagram representing the calculation of DEGs up_regulated and down_regulated in normal and tumor tissues across multiple datasets. DEGs: differentially expressed genes. **C** Heatmap plots illustrating the expression of the selected genes as indicated in (**B**). **D** KEGG pathway analysis of DEGs in normal and tumor tissues. **E** Gene Ontology Biological Processes (GO-BP) term enrichment analysis of DEGs in normal and tumor tissues. **F** Venn diagram representing the calculation of co-expressed genes between up_regulated and EMT core genes. **G** Violin plots illustrate the selected genes according to the 12 EMT core genes, colored by tissue.

To thoroughly investigate the heterogeneity of epithelial cells in BTC, we performed an extensive analysis of cells from both tumors and normal tissues. Initially, differential analysis between normal and tumor tissues identified 1139 up-regulated genes and 715 down-regulated genes in the tumor samples (Fig. 4A). To assess the specificity of these DEGs, we compared them with DEGs from the TCGA database and bulk RNA-seq data (Figure S4A, S4B). This comparison revealed that 198 up-regulated genes and 351 down-regulated genes were unique to the tumor tissues (Fig. 4B, C, Supplementary Table S4).

We then conducted KEGG pathway and GO ontology enrichment analyses on the identified up-regulated and down-regulated genes. The results indicated that the up-regulated genes were predominantly associated with biological processes such as extracellular matrix-receptor interaction, epithelial development, tumor migration, and cell adhesion. These processes are closely linked to tumor growth, invasion, and metastasis. The down-regulated genes were predominantly associated with pathways involved in metabolic processes, such as metabolic pathways, fatty acid degradation, steroid and cholesterol metabolism. This pattern suggests characteristic metabolic reprogramming in tumor cells (Fig. 4D, E, S4C, S4D). These results highlight that EMT-related genes and pathways are significantly up-regulated in BTC, underscoring the critical role of EMT in tumor progression.

We further compared the up-regulated genes with previously identified EMT-related genes, which led to the identification of 12 co-expressed genes (Fig. 4F). These genes displayed significant differential expression in epithelial cells from both tumor and normal tissues (Fig. 4G). This finding underscores the active role of the EMT process in cholangiocarcinoma and highlights the contribution of the EMT population to tumor development. It suggests that tumor-specific EMT genes may facilitate disease progression by engaging in various biological processes. These genes not only enhance our understanding of the molecular mechanisms driving tumor formation and progression but also potentially offer targets for future therapeutic strategies.

### PMEPA1 as a potential EMT marker

**Fig. 5:**
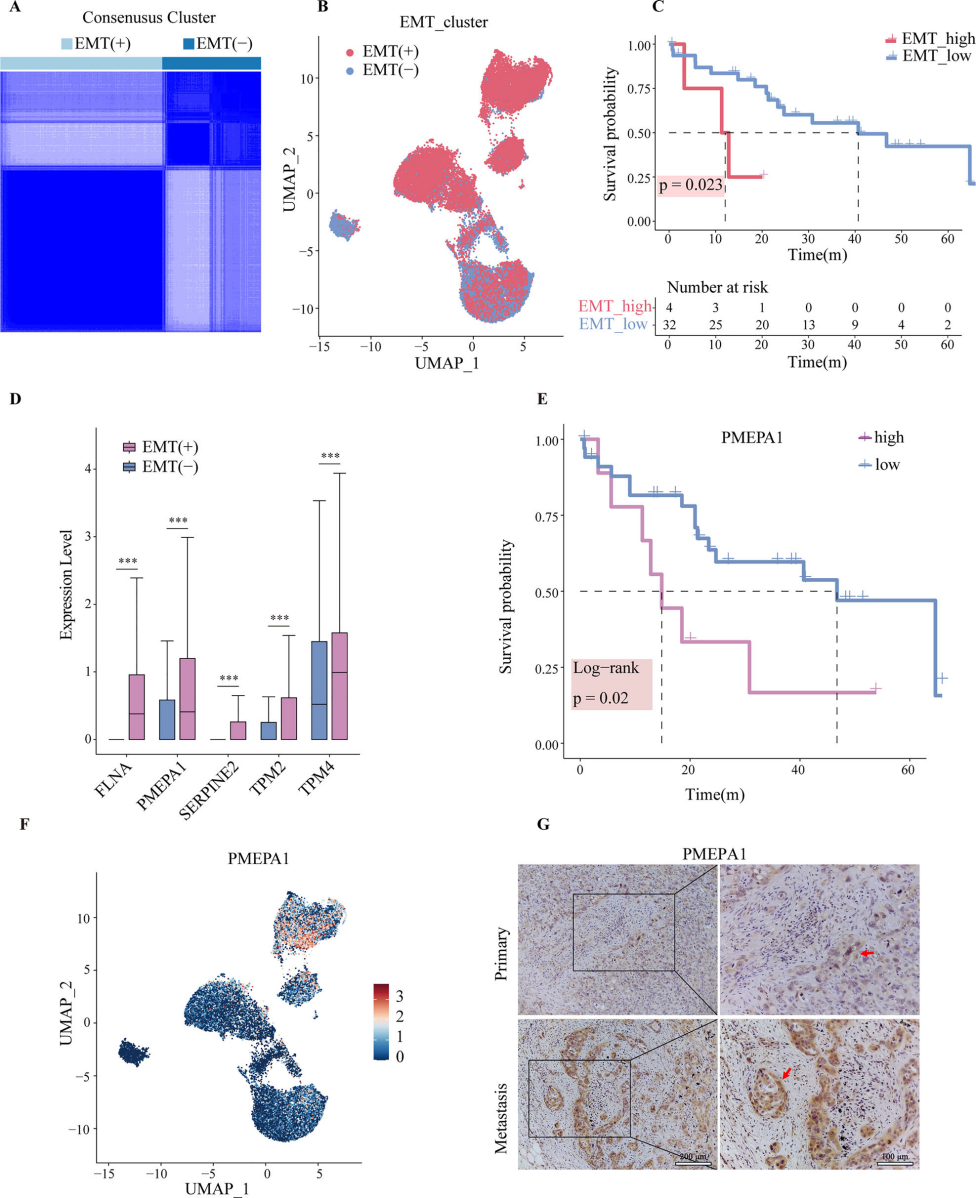
PMEPA1 as a potential EMT marker. **A** Consensus matrix plot based on 51 EMT core genes, representing the proportion of epithelial cell co-occurrence at k = 2, where the distribution nearly reaches its peak. This approach identified BTC cell subtypes, distinguishing between EMT (+) and EMT (−) cells. Dark blue represents a high consensus, whereas white denotes a low consensus. **B** UMAP plot showing the EMT cluster in Epithelial cells. **C** Kaplan–Meier survival curves of patients using the optimal group cut off point with BTC in The Cancer Genome Atlas (TCGA). Patients had scores based on the 51 EMT core genes, with higher EMT scores correlating to poorer prognosis. *p*-values were obtained from 2-sided log-rank tests. **D** Box plots illustrate the varying selected gene expression levels, including FLNA, PMEPA1, SERPINH2, TPM2, TPM4, in EMT (+) and EMT (−) cells. The boxes show the lower quartile, middle quartile and upper quartile. Whiskers represent 1.5 times the interquartile range (IQR). **E** Kaplan–Meier survival curve of PMEPA1 expression using the optimal group cut off point in patients with ICC (TCGA). *p*-values were obtained from 2-sided log-rank tests. **F** UMAP plot showing the expression of PMEPA1 in Epithelial cells. **G** Immunohistochemistry showing the expression level of PMEPA1 in primary and metastatic BTC tissues.

To further investigate the role of EMT in BTC and its correlation with tumor metastasis, we classified epithelial cells using a reference set of 51 EMT-related genes. Consensus clustering analysis identified 18,771 cells as EMT-positive and 11,462 cells as EMT-negative (Fig. 5A, B). We then examined the relationship between EMT levels and survival rates in 36 BTC patients by evaluating EMT levels from the TCGA database. Patients were categorized into high and low EMT expression groups according to their EMT levels. Survival analysis indicated that patients with lower EMT levels had significantly better five-year survival rates (Fig. 5C). This finding underscores the importance of EMT levels as a prognostic indicator for BTC patients. To pinpoint key EMT genes, we conducted differential analyses between EMT-positive and EMT-negative cell subsets. The results identified FLNA, PMEPA1, SERPINE2, TPM2, and TPM4 as genes specifically expressed in EMT-positive cells (Fig. 5D). To explore the potential prognostic value of these genes, we evaluated their survival curves using data from the TCGA database (Figure S5A). Our analysis revealed that high PMEPA1 expression is significantly associated with poor prognosis in BTC patients (Fig. 5E).

Further analysis showed that PMEPA1 expression was notably higher in EMT-positive cells compared to EMT-negative cells (Fig. 5F, S5B). We performed immunohistochemical analysis to compare PMEPA1 expression in carcinoma in situ and lymphatic metastatic cholangiocarcinoma. The results revealed that PMEPA1 expression was significantly higher in lymphatic metastases compared to carcinoma in situ (Fig. 5G). These results indicate that PMEPA1 is not only a crucial molecule in the EMT process but also a promising target for future clinical therapy and detection. Our results highlight the role of EMT in the progression of BTC and suggest that targeting EMT-related genes, particularly PMEPA1, could be a valuable approach for developing therapeutic strategies, especially for patients with high EMT expressions.

### The functional role of PMEPA1 in promoting BTC metastasis

**Fig. 6:**
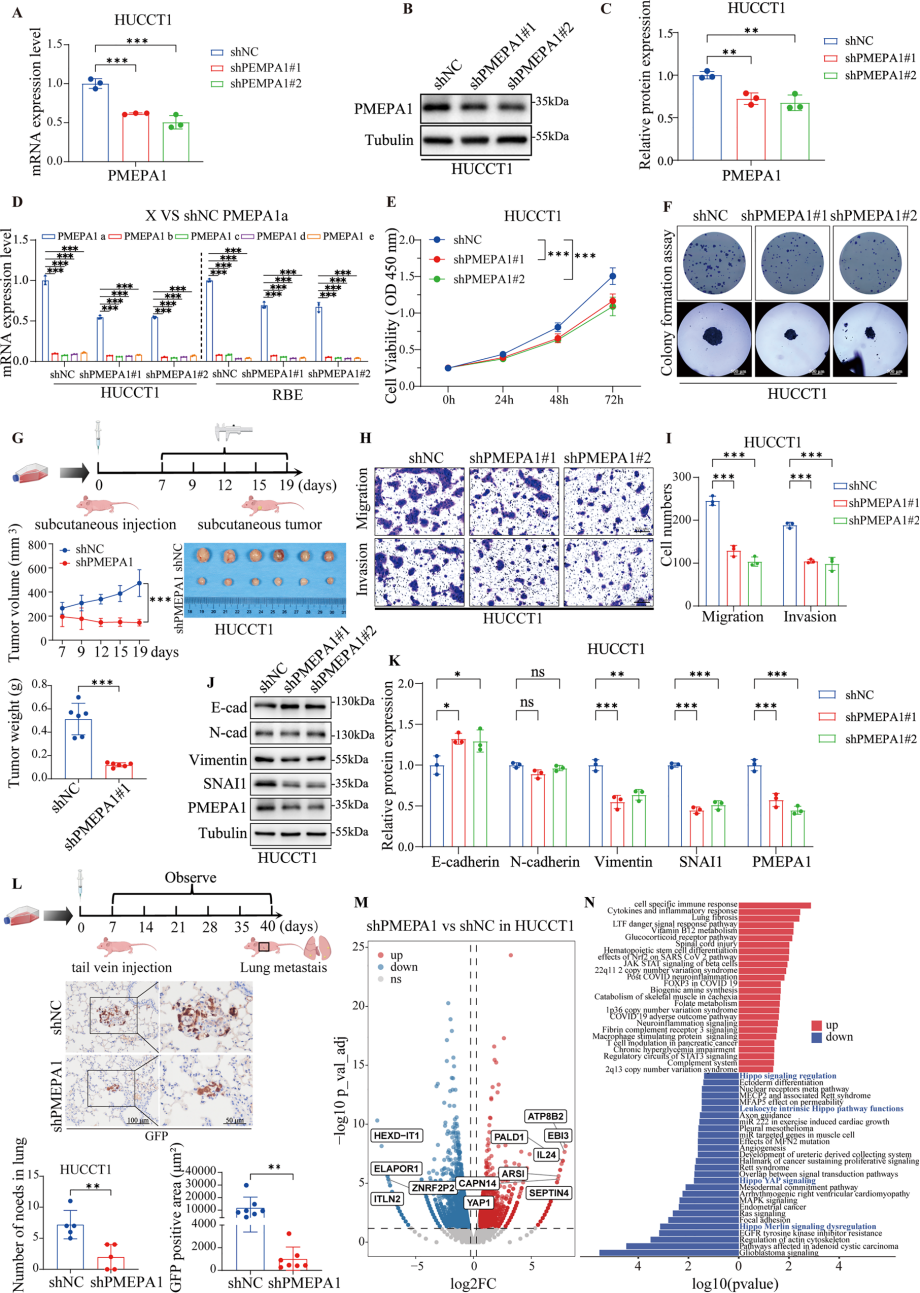
The functional role of PMEPA1 in promoting BTC metastasis. **A** qRT-PCR analysis shows that PMEPA1 mRNA levels are significantly depleted (shPMEPA1) in HUCCT1 compared with shNC. **B**, **C** Western blotting analysis demonstrates PMEPA1 protein levels in shPMEPA1 transfected HUCCT1 cells. The corresponding bar graph quantifies PMEPA1 expression. **D** qRT-PCR analysis that mRNA levels of different PMEPA1 isoforms (a, b, c, d, e) in knockdown HUCCT1 and RBE cell lines. (compared with isoforms a, the *p*-values were all less than 0.05, and the results were obtained from three repeated experiments). **E** Line graph illustrating the growth curves of shNC and shPMEPA1 in HUCCT1. The proliferation rates of both groups at different time points are shown. **F** Colony Formation Assay showing the proliferation of shNC and shPMEPA1 in HUCCT1 (Scale bar = 200 μm). **G** HUCCT1 cells with or without PMEPA1 knockdown were subcutaneously injected into nude mice. Tumor volume and weight were measured after 7, 9, 12, 15, and 19 days. Representative tumor images and quantitative analysis are shown. The number of nude mouse tumor samples was 6. **H** Representative images of HUCCT1 cell migration and invasion observed in the transwell assay (Scale bar = 200 μm). **I** Histogram plots display the cell number of shPMEPA1 and shNC. **J**, **K** Western blot analysis showing E-cadherin, N-cadherin, Vimentin, SNAI1 and PMEPA1 protein levels in shPMEPA1 silenced HUCCT1 compared to shNC. The column diagram showed the expression level of E-cadherin, N-cadherin, Vimentin, SNAI1 and PMEPA1 proteins. **L** HUCCT1 cells expressing shNC or shPMEPA1 were injected into nude mice via tail vein. After 40 days, lung tissues were collected and subjected to GFP immunohistochemistry. Quantification of metastatic nodule number and size confirmed that PMEPA1 knockdown significantly inhibited lung colonization (Scale bar = 100 μm and 50 μm). The number of nude mouse tumor samples was 5. **M** Volcano plots displaying the DEG between shNC and shPMEPA1 in HUCCT1 cells. Blue represents down-regulated genes, red represents up-regulated genes, and gray dots represent no differential expression. **N** Wikipathway analysis illustrates the signaling pathways associated with DEGs following PMEPA1 knockdown. (Red: up regulated pathways, blue: down regulated pathways). Data are presented as mean values with standard deviations. Statistical analysis used the two-sided t-test for comparisons between two groups and one-way ANOVA with Bonferroni correction for comparisons among multiple groups. For CCK-8 assays used two-way ANOVA with Bonferroni correction. (n not significant, *P < 0.05, **P < 0.01, ***P < 0.001, *n* = 3).

Analysis of clinical databases pointed to an oncogenic role for PMEPA1 in BTC. To further investigate its functional impact, we performed PMEPA1 knockdown in HUCCT1 and RBE cells. The knockdown efficiency was confirmed at both mRNA and protein levels (Fig. 6A–C, S6A–S6C). Similarly, successful overexpression of PMEPA1 was validated in these cell lines (Figures S6J, S6K). It is worth noting that five distinct PMEPA1 isoforms (a–e) have been identified in current literature, which may exert diverse biological functions across different tumors or tissue types [40]. Among these isoforms, PMEPA1 a is highly expressed in multiple cancer types, while PMEPA1 b expression is notably decreased or lost in prostate cancer patients [41]. We evaluated the mRNA expression levels of PMEPA1 isoforms a, b, c, d, and e in HUCCT1 and RBE cells (Fig. 6D). The results showed that PMEPA1a exhibited higher expression compared to the other isoforms, suggesting that PMEPA1a may represent the predominant functional isoform in cholangiocarcinoma. Short-term proliferation assays and long-term colony formation assays showed significant differences between control cells and PMEPA1 depleted cells (FigS. 6E, F, S6D, S6E), while PMEPA1 overexpression group were significantly higher than those in the control cells (Figure S6L, S6N). In vivo tumorigenesis, the assays in mice showed that PMEPA1 knockdown significantly reduced tumor volume and weight compared to the control group (Fig. 6G). Moreover, the PMEPA1 knockdown significantly impaired the migratory and invasive abilities of BTC cells (Figs. 6H, I, S6F, S6G), while the overexpression group showed the opposite effect (Figure S6M).

To further investigate the role of PMEPA1 in EMT, we examined changes in the expression of EMT-related markers in HUCCT1 and RBE cell lines. Stable knockdown of PMEPA1 led to upregulation of E-cadherin and downregulation of SNAI1 and Vimentin (Figs. 6J, 6K, S6H, S6I). Conversely, overexpression of PMEPA1 in these cells resulted in reduced E-cadherin levels and increased expression of N-cadherin, SNAI1, and Vimentin (Figure S6O). In addition, we established a lung metastasis model by injecting HUCCT1 cells with stable PMEPA1 knockdown into mice via the tail vein and monitored tumor progression at multiple time points (7, 14, 21, 28, 35, and 40 days). Lung tissues were collected for immunohistochemical analysis 40 days post-injection. The results showed high GFP expression in lung metastatic lesions of the control group, whereas the knockdown group exhibited markedly reduced GFP levels (Fig. 6L). These findings suggest that PMEPA1 is a crucial factor associated with the metastasis of cholangiocarcinoma. Collectively, these data highlight the specific regulatory role of PMEPA1 in BTC cell invasiveness.

To elucidate how PMEPA1 promotes metastasis, we conducted RNA-seq on PMEPA1-knockdown HUCCT1 cells. This revealed 1,345 up-regulated and 4,399 down-regulated genes (Fig. 6M). Wikipathways analysis (Fig. 6N) linked up-regulated genes to immune or inflammatory pathways (e.g., cytokine signaling, complement system), implying PMEPA1 loss enhances anti-tumor immunity. down-regulated genes were enriched in the Hippo signaling pathway, which is known to drive metastasis and therapy resistance in cholangiocarcinoma via YAP activation, suggesting PMEPA1 facilitates progression through Hippo pathway modulation. [42]. Furthermore, additional down-regulated pathways were linked to oncogenesis and cytoskeletal remodeling, processes critical for cell migration, EMT, and invasion. Collectively, our results suggest that PMEPA1 enhances the invasive potential of cholangiocarcinoma primarily through activation of the Hippo-YAP signaling pathway.

### PMEPA1 regulates Hippo-YAP signaling to drive EMT in BTC

In recent years, the Hippo-YAP signaling axis has emerged as a critical regulator of cell proliferation, migration, and EMT across various cancer types [43]. This evolutionarily conserved pathway maintains tissue homeostasis and organ size via a kinase cascade involving MST1/2 and LATS1/2, which phosphorylate and inactivate YAP/TAZ, thereby preventing their nuclear translocation and transcriptional co-activation [44]. Under pathological conditions, such as in malignant tumors, disruption or inactivation of Hippo signaling results in sustained activation and nuclear accumulation of YAP/TAZ, promoting the transcription of oncogenic targets including CYR61 and SNAI1, enhancing tumor cell invasiveness and metastatic potential [45–47]. Notably, aberrant activation of the Hippo-YAP pathway has been implicated in the progression of BTC, where elevated YAP1 expression is closely associated with poor clinical outcomes [48–50].

**Fig. 7:**
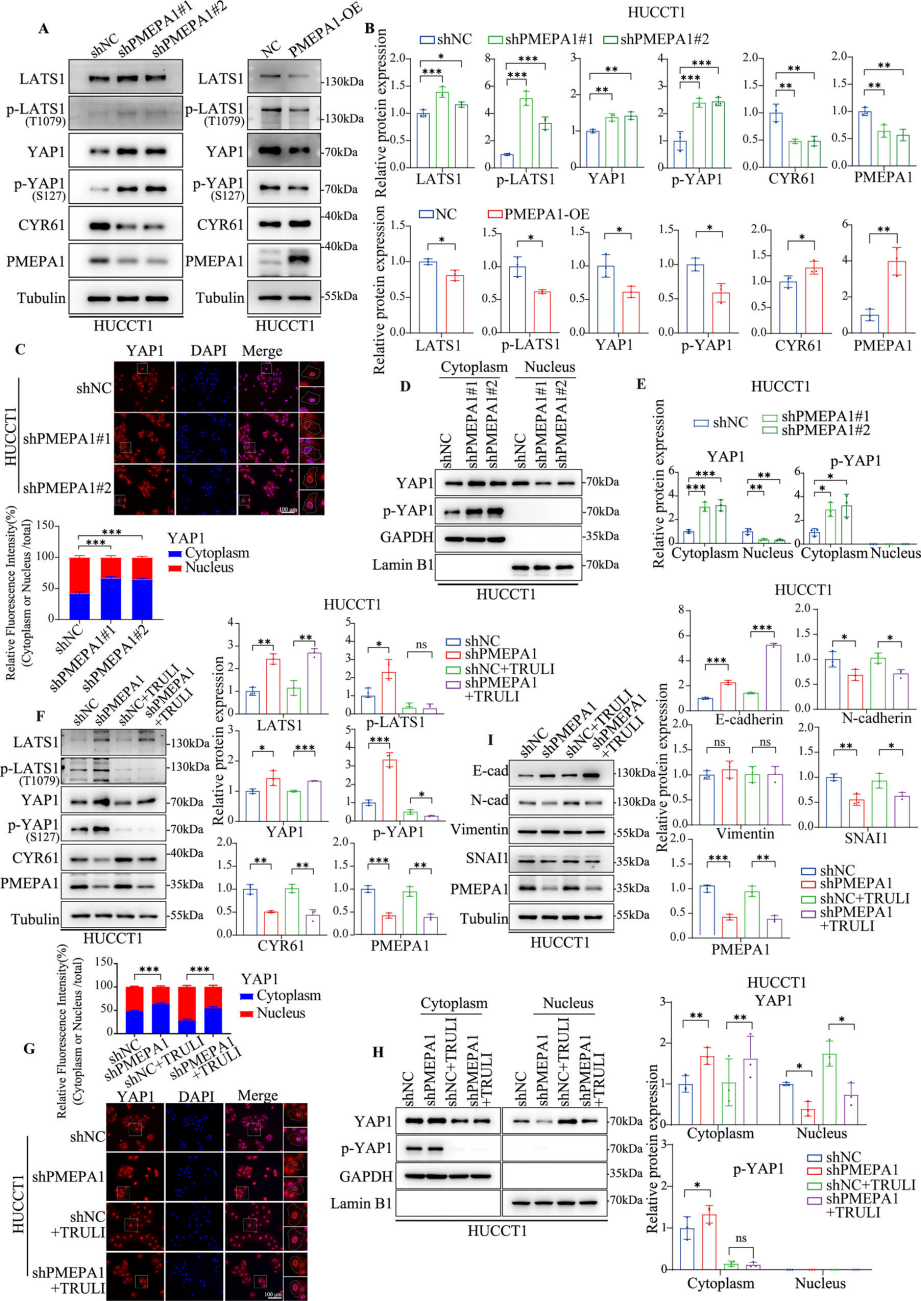
PMEPA1 regulates Hippo-YAP signaling to drive EMT in BTC. **A**, **B** Western blotting analysis of the expression levels of Hippo pathway related marker genes and PMEPA1 protein in HUCCT1 cells, and bar graphs showing the statistical analysis of expression levels. **C** Immunofluorescence staining images of YAP1 (red) showing its cellular localization in HUCCT1 cells. The nucleus is stained with DAPI (blue). The relative fluorescence intensity of YAP1 in the cytoplasm or nucleus was quantified and presented as a ratio to the total cellular fluorescence (Scale bar = 100 μm). **D**, **E** Western blotting analysis of YAP1 and p-YAP1 expression levels and corresponding statistical bar graph in HUCCT1 cells. Compared to the control group, nuclear expression was significantly reduced. GAPDH was used as a cytoplasm normalization control, while Lamin B1 was employed as a nucleus normalization control. **F** Western blotting analysis of the expression levels of Hippo pathway-related marker genes and PMEPA1 protein in HUCCT1 cells, and bar graphs showing the statistical analysis of expression levels. **G** Immunofluorescence staining images of YAP1 (red) showing its cellular localization in HUCCT1 cells. The nucleus is stained with DAPI (blue). The relative fluorescence intensity of YAP1 in the cytoplasm or nucleus was quantified and presented as a ratio to the total cellular fluorescence (Scale bar = 100 μm). **H** Western blotting analysis of YAP1 and p-YAP1 expression levels and corresponding statistical bar graph in HUCCT1 cells. Compared to the control group, nuclear expression was significantly reduced. GAPDH was used as a cytoplasm normalization control, while Lamin B1 was employed as a nucleus normalization control. **I** Western blotting analysis of the expression levels of EMT-related marker genes and PMEPA1 protein in HUCCT1 cells, and bar graphs showing the statistical analysis of expression levels. Data are presented as mean values with standard deviations. Statistical analysis used the two-sided t-test for comparisons between two groups and one-way ANOVA with Bonferroni correction for comparisons among multiple groups. (ns not significant, *P < 0.05, **P < 0.01, ***P < 0.001, *n* = 3).

Based on our prior findings, we hypothesized that PMEPA1 acts as an upstream regulator of the Hippo-YAP pathway. To test this, we examined key Hippo pathway components in PMEPA1-manipulated BTC cells by Western blot. Silencing PMEPA1 markedly increased the protein levels of LATS1 and its phosphorylated form (p-LATS1), as well as the phosphorylation of YAP1 (Figs. 7A, 7B, S7A, S7B). Conversely, PMEPA1 overexpression produced the opposite effect. These alterations suggest that PMEPA1 deficiency promotes YAP1 cytoplasmic retention and inactivation. Consistently, the expression of CYR61, a canonical YAP target gene, was down-regulated, further supporting PMEPA1 as a positive regulator of YAP signaling. To directly visualize YAP1 localization, we performed subcellular fractionation and immunofluorescence staining. The latter revealed a clear retention in cytoplasmic YAP1 in two independent PMEPA1-knockdown cell lines (Fig. 7C, S7C). This observation was confirmed by Western blot analysis of fractionated lysates, which showed a pronounced increase of YAP1 in the cytoplasm and a concomitant decrease in the nucleus upon PMEPA1 depletion (Figs. 7D, 7E, S7D, S7E). Collectively, our studies establish that PMEPA1 may suppress Hippo pathway activity, thereby preventing YAP1 cytoplasmic sequestration and sustaining its transcriptional output.

To further delineate the mechanistic link between PMEPA1 and the Hippo-YAP axis, we performed rescue experiments with TRULI, a specific ATP-competitive inhibitor of LATS1/2 kinases that blocks LATS-mediated YAP1 phosphorylation and promotes its nuclear function. Treatment of control (shNC) HUCCT1 and RBE cells with 10 μM TRULI (HY-138489) effectively suppressed YAP1 phosphorylation without altering total LATS1 or YAP1 levels (Fig. 7F, S7F). Crucially, in PMEPA1-knockdown cells, TRULI treatment potently reduced levels of p-LATS1 and p-YAP1. Furthermore, this pharmacological inhibition of LATS1/2 not only reversed the cytoplasmic retention of YAP1 induced by PMEPA1 knockdown (Figs. 7G, 7H, 7I, S7G, S7H, S7I) but also rescued the associated EMT phenotype, as evidenced by downregulation of N-cadherin and SNAI1 and upregulation of E-cadherin. These results indicate that PMEPA1 promotes EMT in BTC at least in part by modulating the Hippo-YAP pathway. Collectively, our findings support a model wherein PMEPA1 enhances YAP1 nuclear function by suppressing LATS1/2 kinase activity, thereby facilitating EMT and driving the invasive and metastatic properties of BTC cells.

### Screening of EMT-targeted therapeutic drugs for BTC

**Fig. 8:**
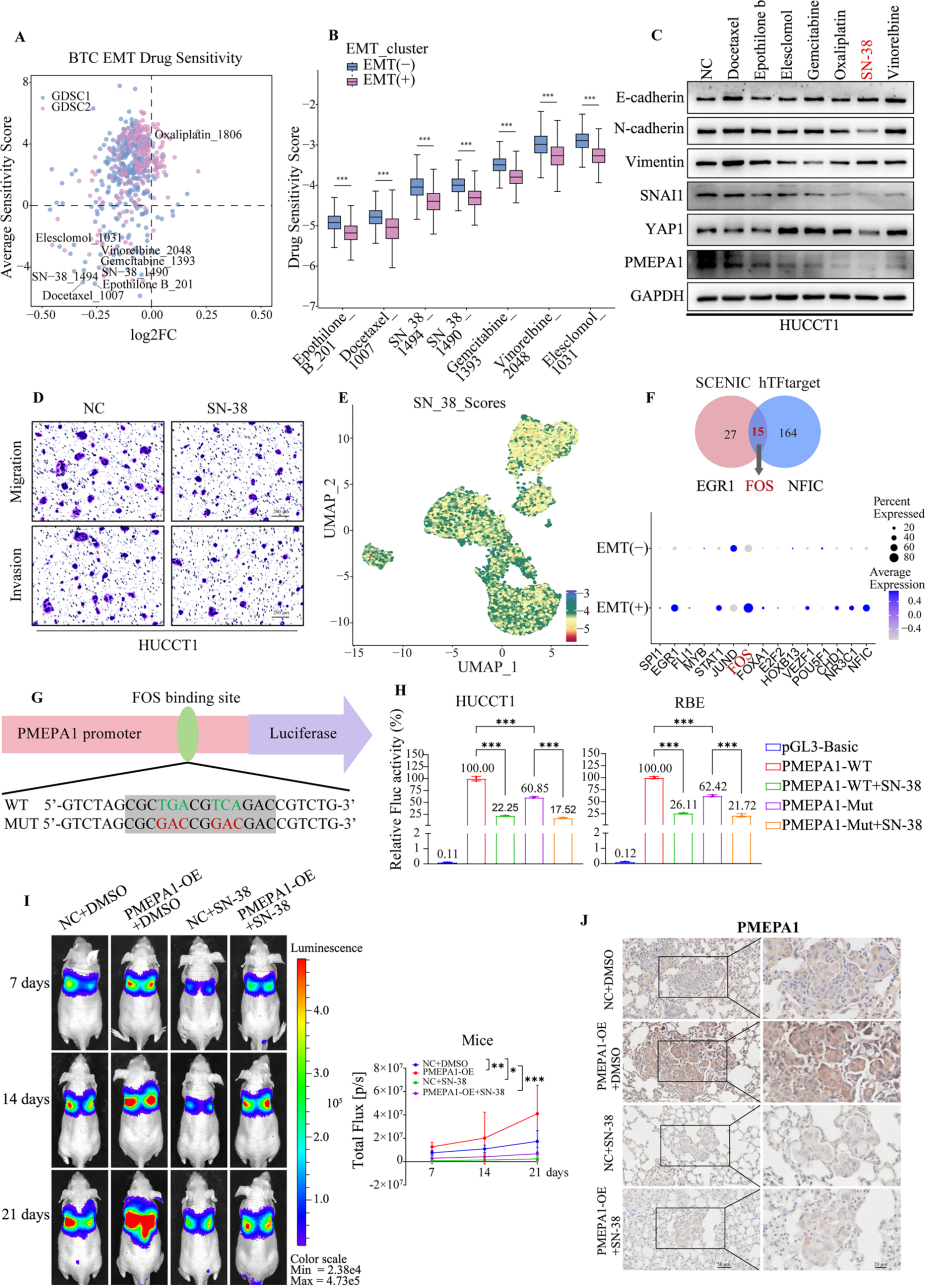
Screening of EMT-targeted therapeutic drugs for BTC. **A** Scatter plot of EMT cluster cell drug sensitivity predictions. **B** Box plots depicting variations in drug expression levels, including epothilone b, docetaxel, SN-38, gemcitabine, vinorelbine, and elesclomol, between the EMT (+) and EMT (−) groups based on the GDSC database. The boxes show the lower quartile, middle quartile and upper quartile. Whiskers represent 1.5 times the interquartile range (IQR). **C** Western blotting showing the comparison of E-cadherin, N-cadherin, Vimentin, SNAI1, YAP1 and PMEPA1 expression level in HUCCT1 after treatment with treated with seven different drugs. **D** Transwell assays revealing changes in HUCCT1 after treatment with SN-38 (Scale bar= 200 μm). **E** UMAP plot showing the SN-38 scores for EMT (+) epithelial cells from the GDSC database. Lower scores indicate better drug effects. **F** Venn diagram shows 15 common transcription factors shared by SCENIC and hTFtarget. Dot plot shows the expression levels of 15 transcription factors in the EMT subgroups. The percentage of cells expressing the marker gene is represented by dot size, while dot color denotes the average expression level. **G** Schematic representation of wild-type and mutant FOS binding motifs in PMEPA1 region. **H** The relative luciferase activity of luciferase reporter plasmids containing wild-type or mutant PMEPA1 promoter sequences or treated with SN-38 was detected in HUCCT1 and RBE cells. **I** In vivo bioluminescent images and quantification of NC + DMSO, PMEPA1-OE + DMSO, NC + SN-38 and PMEPA1-OE + SN-38 derived nude mice lung metastasis model at the indicated time points. **J** Immunohistochemical detection of the expression level of PMEPA1 in the metastatic lung tissues of mice (Scale bar = 50 μm and 20 μm). Data are presented as mean values with standard deviations. Statistical analysis used one-way ANOVA with Bonferroni correction for comparisons among multiple groups. The fluorescence imaging of mic used two-way ANOVA with Bonferroni correction. The number of nude mouse tail vein lung metastasis model samples was 5. (ns not significant, *P < 0.05, **P < 0.01, ***P < 0.001, *n* = 3).

Given that EMT is a primary mechanism driving invasion and metastasis in BTC, identifying drugs that effectively inhibit this process is crucial for improving patient outcomes. To this end, by leveraging the GDSC1 and GDSC2 drug databases with the expression matrix of the EMT subsets in epithelial cells, we conducted predictions of their tumor cell drug sensitivity. Initially, we classified cells into EMT-positive and EMT-negative groups and identified drugs specifically targeting EMT that are used in clinical practice. Drugs with low Log2 Fold Change values and low average sensitivity scores were considered as predicted candidate drugs for targeting EMT in BTC. Based on this analysis, we predicted that drugs such as epothilone B, docetaxel, and SN-38 could be effective in treating BTC (Fig. 8A). Notably, chemotherapy drugs like epothilone B, docetaxel, SN-38, gemcitabine, vinorelbine and elesclomol received high efficacy scores (Fig. 8B). Additionally, we observed that the current first-line therapy for BTC, Oxaliplatin, may exhibit notable resistance (Figure S8A). As shown in Supplementary Table S8, HUCCT1 and RBE cells were treated with docetaxel (0.05 μM, HY-B0011), epothilone B (1 μM, HY-17029), elesclomol (0.5 μM, HY-12040), gemcitabine (0.2 μM, HY-17026), oxaliplatin (50 μM, HY-17371), SN-38 (1 μM, HY-13704) or Vinorelbine ditartrate (0.06 μM, HY-12053A). To validate our predictions, we selected the top six predicted drugs for EMT treatment, along with oxaliplatin, for experimental testing. We assessed changes in EMT-related markers in response to these drugs. As shown in Fig. 8C and Figure S8C, treatment with SN-38 led to an upregulation of E-cadherin expression and a concomitant downregulation of N-cadherin, SNAI1, and Vimentin in HUCCT1 and RBE cells. Cell invasion and migration assays revealed that SN-38 drugs significantly reduced the migratory and invasive capabilities of HUCCT1 and RBE cells (Fig. 8D, S8D, S8E). Notably, the expression levels of PMEPA1 and YAP1 were also significantly reduced. UMAP analysis of epithelial cells indicated that SN-38 strongly inhibited EMT cells (Fig. 8E, S8B). SN-38 is the active metabolite of the chemotherapeutic agent irinotecan and functions by inhibiting DNA topoisomerase I, thereby disrupting DNA and RNA synthesis and inducing frequent DNA single-strand breaks. To further elucidate the regulatory mechanism by which SN-38 regulates PMEPA1, we utilized the SECINE algorithm and the hTFtarget database to predict upstream transcription factors of PMEPA1, identifying 15 common candidates. Among these, transcription factors FOS were found to be highly expressed in the EMT-positive subcluster (Fig. 8F). Using the JASPAR database, we further predicted the binding site (CGCTGACGTCAGAC) of transcription factor FOS in the PMEPA1 promoter region (Fig. 8G). To validate this prediction, a fragment of the PMEPA1 promoter (−2000 - +500 bp) containing the wild-type (WT) or a mutated (Mut) FOS binding site was cloned into the pGL3-Basic luciferase reporter vector. The WT and Mut constructs were co-transfected with the pRL-TK control plasmid into HUCCT1 and RBE cells, followed by treatment with SN-38 (1 μM). Dual luciferase reporter assays revealed that mutation of the FOS binding site markedly decreased the promoter activity of PMEPA1 compared with the WT construct, indicating that FOS enhances PMEPA1 transcriptional activation. Notably, SN-38 treatment further reduced luciferase activity, with a more pronounced inhibitory effect observed in the Mut group (Fig. 8H). These findings indicate that SN-38 may inhibit the metastasis of biliary tract cancer by downregulating a PMEPA1-FOS pathway.

To further verify the inhibitory effect of SN-38 on PMEPA1-mediated lung metastasis in vivo, we established a lung metastasis model by injecting nude mice with HUCCT1 cells stably overexpressing PMEPA1 tagged with luciferase via the tail vein. One-week post-injection, mice in both the NC and PMEPA1-OE groups were randomly divided into two subgroups, receiving either SN-38 treatment group or DMSO solvent via intraperitoneal injection. In vivo bioluminescence imaging was performed at 7, 14, and 21 days after drug administration (Fig. 8I). The results showed that the bioluminescence signal in PMEPA1-overexpressing mice was markedly stronger than that in the NC group. Notably, the time-dependent decrease in bioluminescence signal reflected inhibited tumor growth in the treatment group. As shown in Fig. 8J, S8F and S8G, Immunohistochemistry and Western blot analyses further indicated that, relative to the PMEPA1 overexpression group, SN-38 treatment led to a notable reduction in metastatic lesion size, decreased PMEPA1 expression. In summary, our findings suggest that PMEPA1 may serve as a potential molecular target of SN-38, likely influencing the EMT process through modulation of the Hippo signaling pathway. However, the precise mechanisms remain to be elucidated in future studies. Moreover, combinatorial treatment strategies involving YAP/TAZ inhibitors and targeted agents may further enhance the therapeutic efficacy against cholangiocarcinoma, particularly by reinforcing the inhibition of EMT-associated pathways [49].

Therefore, EMT-positive cells and PMEPA1 may serve as promising targets for future immunotherapeutic interventions. In conclusion, our evaluation of targeted therapies in BTC highlights the critical role of EMT inhibition and provides novel insights into the development of effective treatment strategies for BTC.

## Discussion

Single-cell analyses have substantially advanced our understanding of biliary tract cancer (BTC), particularly intrahepatic cholangiocarcinoma (iCCA). For instance, Zhang *et al*. identified a subset of fibroblasts—termed vCAF—that promote EZH2 expression in iCCA via IL-6 secretion, thereby enhancing tumor malignancy [51]. In another study, single-cell analysis of 14 untreated iCCA tumors and paired adjacent tissues led to the identification of two molecular subtypes, iCCA^phl^ and iCCA^pps^ [52]. We systematically analyzed scRNA-seq data from 47 BTC samples, constructing a relatively comprehensive single-cell atlas of BTC that includes a wide range of cell types such as epithelial and immune cells, thereby advancing current knowledge of BTC. We identified distinct EMT subpopulations and thoroughly investigated their functional roles and molecular heterogeneity, leading to the identification of EMT-related genes implicated in tumor progression and metastasis.

In recent years, numerous studies have highlighted the importance of EMT heterogeneity and subtype distinctions. Studies on bladder and liver cancers have shown that different tumor subtypes exhibit distinct EMT features and gene expression profiles, which are closely associated with tumor aggressiveness and patient prognosis [53, 54]. EMT scoring has also emerged as a valuable tool for predicting metastatic potential [12]. In esophageal squamous cell carcinoma, EMT scoring successfully identified EMT subtypes and key EMT signature genes [16]. These studies collectively underscore the importance of EMT heterogeneity in cancer progression, supporting our focus on EMT subpopulations in BTC. Although our study included a relatively large number of samples, the latent onset of BTC and challenges in sample collection still limit a more comprehensive understanding of its complex biology. Future studies should broaden the sources of BTC samples to achieve a more complete understanding of their pathogenesis and progression.

BTC is characterized by high invasiveness and heterogeneity, features closely linked to EMT [55, 56]. Although these studies have made progress in exploring the functions of EMT-related genes, in-depth mechanistic studies on EMT-associated signature molecules are still lacking. While previous studies have identified EMT-related genes, the underlying molecular mechanisms remain inadequately explored. Our findings highlight PMEPA1 as a key regulator of tumor phenotypic transition and invasive behavior. RNA-seq analysis revealed that PMEPA1 modulates a regulatory network centered on the Hippo signaling pathway, a central coordinator of tissue growth and tumor development [57]. We further elucidated how PMEPA1 promotes BTC metastasis through Hippo-YAP signaling, though the precise mechanisms by which PMEPA1 engages this axis during EMT and metastasis warrant further investigation.

Fibrosis plays a significant role in BTC progression and presents a major clinical challenge [58, 59]. It promotes tissue stiffness, fostering a microenvironment conducive to tumor invasion and therapy resistance [60]. In our data, several genes associated with EMT, such as TIMP1 and MMP1, were also implicated in fibrosis [61, 62]. Moreover, TGF signaling, a major regulator of EMT, is a pivotal driver of fibrosis [63]. Whether these genes directly contribute to fibrotic progression in BTC remains to be determined. PMEPA1 has been implicated in multiple cancer types, such as lung, breast, and prostate cancer, where it exerts context-dependent oncogenic or tumor-suppressive functions via distinct mechanisms [17, 64, 65]. However, its transcriptional regulation in BTC has not been well characterized.

Our study provides preliminary evidence that PMEPA1 regulates EMT in BTC cells via the Hippo-YAP axis. We propose that PMEPA1 inhibits LATS1 kinase activity, facilitating YAP1 nuclear translocation and subsequent activation of EMT-related genes, thereby promoting metastasis. In this model, high PMEPA1 expression inactivates the Hippo pathway, enhancing YAP1-driven transcription, whereas PMEPA1 knockdown restores Hippo-mediated YAP1 suppression and reduces EMT phenotypes. This model is consistent with reports in glioma, where the PMEPA1a isoform promotes NEDD4-mediated ubiquitination and degradation of LATS1, thereby potentiating YAP/TAZ signaling [66].

Given the key regulatory role of PMEPA1 in the Hippo-YAP pathway, the targeted inhibition of PMEPA1 could be a novel therapeutic strategy for BTC. Additionally, YAP/TAZ inhibitors such as verteporfin have been shown to effectively suppress YAP1 activity [67]. Combining PMEPA1 targeted interventions with YAP inhibitors may enhance anti-tumor efficacy. Although our study revealed the role of PMEPA1 in modulating the Hippo-YAP axis, its precise molecular mechanisms remain to be further elucidated. Moreover, regulation of the Hippo pathway is highly complex and may involve additional cofactors or interacting partners. For example, PMEPA1 may influence or integrate with other signaling pathways, such as TGFB signaling, to indirectly regulate the Hippo-YAP axis [68, 69]. These potential synergistic and cross-regulatory mechanisms have yet to be identified and warrant further investigation. PMEPA1 may have multiple functional targets in cells, and whether it contributes to BTC progression through additional mechanisms remains unknown. These open questions highlight the need for more comprehensive studies to fully elucidate PMEPA1 in BTC.

In the field of precision oncology, molecular classification of tumor subtypes is essential for developing personalized treatment strategies. We classified BTC cells into EMT-positive and EMT-negative groups and evaluated potential therapeutic agents targeting EMT. The experiment confirmed that SN-38 could effectively reduce the EMT level of BTC through its association with the downregulation of a PMEPA1-FOS signaling pathway and inhibit cell migration and invasion. Although we did not perform Co-IP or ChIP assays to demonstrate their direct binding, our data strongly suggest that SN-38 may inhibit cholangiocarcinoma metastasis by affecting PMEPA1 transcription. In the vivo experiments, SN-38 treatment significantly reduced the lung metastasis, and this effect was particularly evident in the background of PMEPA1 overexpression, further confirming that PMEPA1 is a potential target for SN-38. Although these drugs showed enhanced therapeutic efficacy, they also have limitations. For example, gemcitabine—currently a first-line treatment for BTC—often leads to drug resistance over time in some patients [70]. Therefore, exploring combination treatment strategies is critical to enhancing therapeutic outcomes.

Recent studies have shown that combining immune checkpoint inhibitors, such as pembrolizumab, with gemcitabine and cisplatin significantly improves overall survival in patients with metastatic BTC [71]. This finding highlights the clinical potential of EMT-related genes such as PMEPA1 as therapeutic targets. Future research should further focus on EMT-targeted therapeutic strategies to improve BTC treatment outcomes. Our findings provide new theoretical foundations for BTC molecular subtyping, precision therapy, and the optimization of combination treatment strategies. In this study, we employed multi-omics bioinformatics analysis techniques, utilizing extensive datasets to thoroughly investigate the complex role of EMT in BTC. We specifically explored the molecular mechanisms by which PMEPA1 activates EMT in BTC progression, aiming to identify potential warning markers for BTC metastasis. Our results demonstrate not only the feasibility of classifying BTC patients based on EMT levels but also underscore its clinical significance. Additionally, we identified potential candidate drugs for BTC patients with high EMT expression, which may enhance their treatment outcomes.

## Supplementary information

Supplementary figures and tables

Unedited blot and gel images

## Data Availability

The original sequencing data in this study can be obtained from the corresponding author. The other supplementary data used in this study were retrieved from the GEO database (https://www.ncbi.nlm.nih.gov/geo/) and UCSC Xena (https://xena.ucsc.edu/). The accession numbers for these datasets are provided in Supplementary Table S1.
