# Supplementary material for: PMEPA1 modulates YAP1 nuclear translocation to disrupt EMT subtypes and promote metastasis in Biliary tract cancer
Source: Cell Death Dis. 2026 Apr 3;17(1):449. doi: 10.1038/s41419-026-08684-3 (PMC13172513; doi:10.1038/s41419-026-08684-3)
Supplement: Supplementary file 1 — Supplementary figures and tables [file 41419_2026_8684_MOESM1_ESM.docx]

**Supplementary Information**

**PMEPA1 modulates YAP1 nuclear translocation to disrupt EMT subtypes and promote metastasis in Biliary tract cancer**

Wenwen Xu^1+, 2+, 3+^, Chaoqun Ma^1+^, Pin Li^4^, Xinxing Lyu^2^, Ruitang Xu^2^, Kaiwen Sheng^2^, Ting Zhang^2^, Haoqiang Sun^2^, ZhaoXiang Zhang^2^, Ziyi Wang^2^, Hongguang Li^1*^, Dianhao Guo^2*^ and Shuhong Huang^2*^

^+^ These authors contributed equally to this work.

**Running Title**

PMEPA1 regulates YAP1 to promote BTC metastasis

^*^ **Corresponding author:**

Shuhong Huang: [shuhonghuang@sdfmu.edu.cn](mailto:shuhonghuang@sdfmu.edu.cn), School of Clinical and Basic Medical Sciences, Shandong First Medical University & Shandong Academy of Medical Sciences, Jinan, Shandong, 250117, China.

Dianhao Guo: dhguo@sdfmu.edu.cn, School of Clinical and Basic Medical Sciences, Shandong First Medical University & Shandong Academy of Medical Sciences, Jinan, Shandong, 250117, China.

Hongguang Li: doctorlihg@163.com, Department of Hepatobiliary Surgery, Shandong Provincial Hospital Affiliated to Shandong First Medical University, Jinan, Shandong,250021, China

**Author emails:**

Wenwen Xu: [xuww1709@163.com](mailto:xuww1709@163.com), Chaoqun Ma: [medkaomcq@qq.com](mailto:medkaomcq@qq.com), Pin Li: [lipinsdu@foxmail.com](mailto:lipinsdu@foxmail.com), Xinxing Lyu: xxlv@sdfmu.edu.cn, Ruitang Xu: [xrt1942644109@163.com](mailto:xrt1942644109@163.com), Kaiwen Sheng: [kaiwen737@163.com](mailto:kaiwen737@163.com), Ting Zhang: Zt980522@163.com, Haoqiang Sun: [sunhaoqiang0925@163.com](mailto:sunhaoqiang0925@163.com), ZhaoXiang Zhang: [1638668921@qq.com](mailto:1638668921@qq.com), Ziyi Wang: 19563486087@163.com.

**Author affiliations:**

^1^ Department of Hepatobiliary Surgery, Shandong Provincial Hospital Affiliated to Shandong First Medical University, Jinan, Shandong, 250021, China.

^2^ School of Clinical and Basic Medical Sciences, Shandong First Medical University & Shandong Academy of Medical Sciences, Jinan, Shandong, 250117, China.

^3^ College of Life Sciences, Shandong Normal University, Jinan, Shandong, 250358, China.

^4^ School of Basic Medical Sciences, Cheeloo College of Medicine, Shandong University, Jinan, Shandong, 250012, China.

**Supplementary Figures legends.**

**Supplementary Tables.**

**Supplementary Figure and Figure legends**

**Supplementary Fig. S1 Heterogeneity in biliary tract cancer.** (A) UMAP plot of the 159,713 cells in BTC, colored by sample. Each dot denotes a single cell. (B) UMAP plots illustrate key marker genes to identify cell types, including B cell (CD79A), cycling cell (MKI67), DC (CLEC10A), Endothelial (VWF), Epithelial (EPCAM), Fibroblast (ACTA2), Macrophage (CD163), Mast cell (TPSAB1), Monocyte (S100A8), T cell (CD3E). (C) Stack histogram shows the proportion of 10 cell types in each sample, colored by cell type.


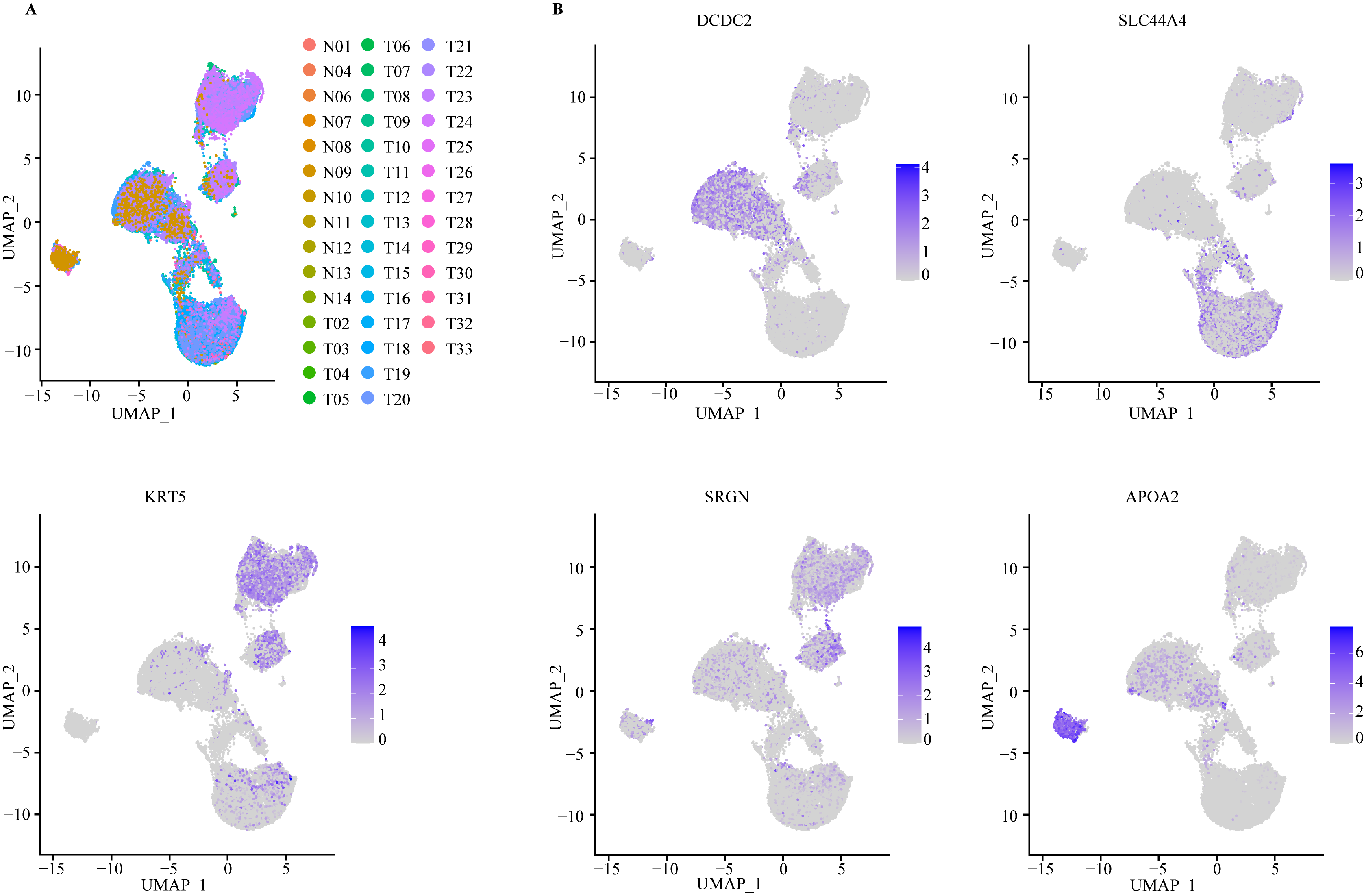


**Supplementary Fig. S2 Epithelial cell heterogeneity in BTC.** (A) UMAP plot of epithelial cells, colored by sample. Each dot denotes a single cell. (B) UMAP plots illustrate key marker genes to identify cell types, including Epi1 (DCDC2), Epi2 (SLC44A4), Epi3 (KRT5), Epi4 (SRGN), Epi5 (APOA2).


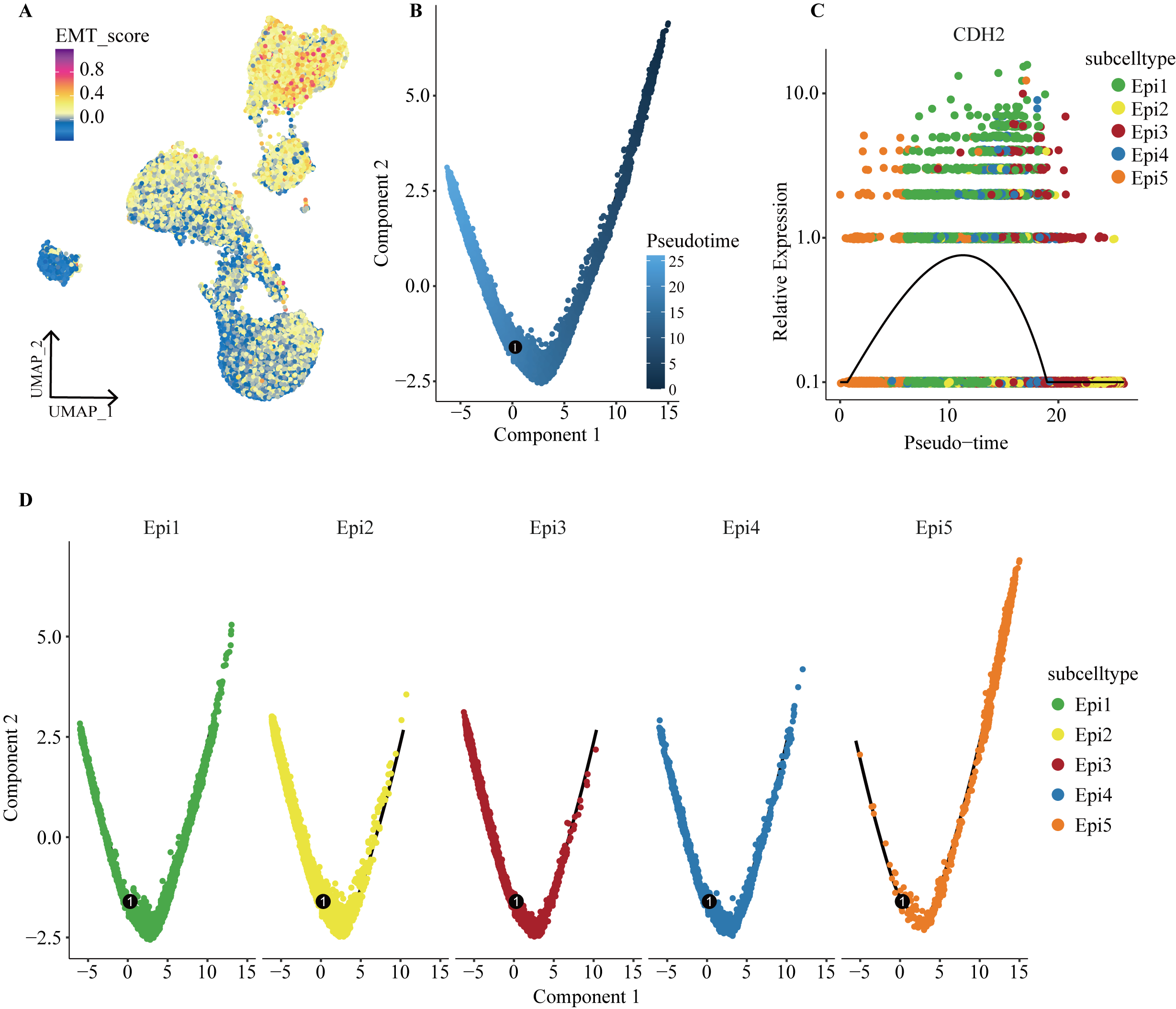


**Supplementary Fig. S3 Pseudotime trajectory analysis in epithelial cell clusters.** (A) The distribution of signature scores of 51 EMT genes of Epithelial shown UMAP plot. Each dot denotes a single cell. (B) Trajectory plot of pseudotime. Each point indicates a single cell. (C) Temporal variation of known mesenchymal marker (CDH2). Each point indicates a single cell, and color represents epithelial cell subtype. (D) Trajectory plot of Epithelial cell subtypes, colored for pseudotime. Each point indicates a single cell.


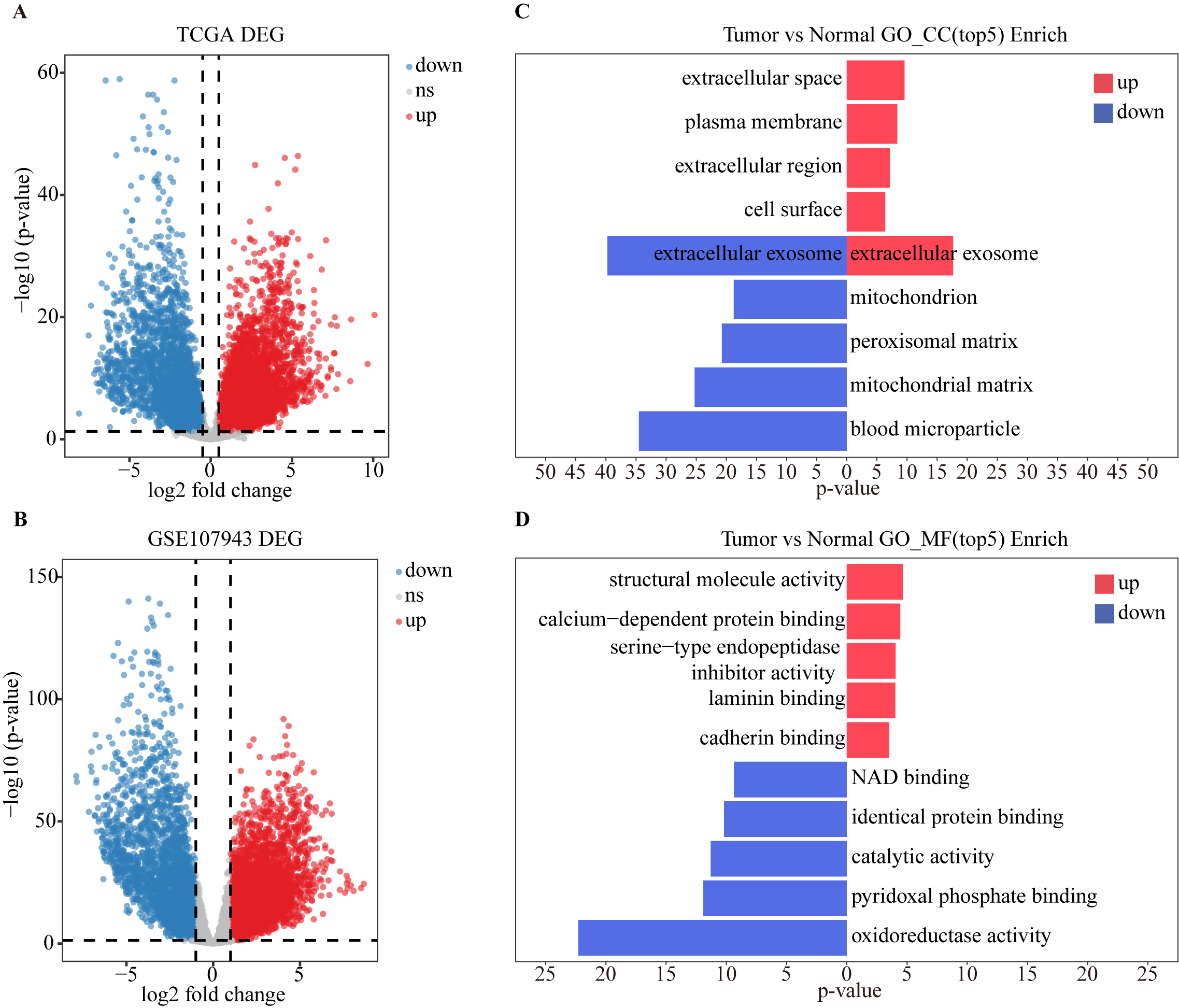


**Supplementary Fig. S4 Differential genes in BTC sample.** (A) Volcano plots displaying the DEGs between Normal and Tumor in BTC from the TCGA database. (B) Volcano plots displaying the DEGs between Normal and Tumor from GSE107943 dataset. Each dot denotes one gene, demonstrating the representative DEGs. Each dot denotes one gene, demonstrating the representative DEGs. Log2FoldChange <−1 represents differentially down_regulated genes, Log2FoldChange >1 represents differentially up_regulated genes, and gray dots represent genes with no differential expression. DEGs: differentially expressed genes. (C) Gene Ontology Cellular Component (GO-CC) term enrichment analysis of intersecting DEGs in Normal and Tumor. (D) Gene Ontology Molecular Function (GO-MF) term enrichment analysis of intersecting DEGs in Normal and Tumor.

**Supplementary Fig. S5 Screening of BTC EMT marker.** (A) Kaplan–Meier survival curve of CAPG, CTHRC1, DKK1, FLNA, ITGA2, LACM2, MMP1, PDLIM4, SERPINE2, TPM2 and TPM4 expression using the optimal group cut-off point in patients with ICC (from TCGA). p values were obtained from 2-sided log-rank tests. (B) UMAP plot showing the expression of CTHRC1, DKK1, FLNA, MMP1, TPM2, in Epithelial cell. Each point indicates a single cell.

**Supplementary Fig. S6 The functional role of PMEPA1 in promoting BTC metastasis.** (A) qRT-PCR analysis shows that PMEPA1 mRNA levels are significantly depleted (shPMEPA1) in RBE compared with shNC. (B, C) Western blot analysis shows that PMEPA1 protein levels are significantly reduced in shPMEPA1 transfected in RBE. The column diagram shows the expression level of PMEPA1. (D) The line graph illustrates the growth curves of shNC and shPMEPA1 in RBE, showing the proliferation rates of both groups at different time points. (E) Histogram plots showed the colony formation assay cell number of shPMEPA1 and shNC in HUCCT1. (F, G) Representative images show the results of the transwell migration and invasion assay in RBE. Histogram plots showed the number of shPMEPA1 and shNC controls. (H, I) Western blot analysis showed E-cadherin, N-cadherin, Vimentin, SNAI1 and PMEPA1 protein levels in the shPMEPA1 silenced RBE compared with the corresponding controls. The column diagram showed the expression of E-cadherin, N-cadherin, Vimentin, SNAI1 and PMEPA1 protein. (J) qRT-PCR analysis showing that PMEPA1 mRNA levels in HUCCT1 and RBE cells. (K) Western blot analysis of PMEPA1 protein levels in HUCCT1 and RBE cells transfected with PMEPA1-overexpression plasmid. (L) Line graph illustrating the growth curves of NC and PMEPA1-OE in HUCCT1 and RBE. The proliferation rates of both groups at different time points are shown. (M) Representative images of HUCCT1 and RBE cell migration and invasion observed in the transwell assay (Scale bar= 200 μm). Histogram plots display the cell number of NC and PMEPA1-OE. (N) Colony Formation Assay showing the proliferation of NC and PMEPA1-OE in HUCCT1 and RBE (Scale bar= 200 μm). Histogram plots show the number of NC and PMEPA1-OE. (O) Western blot analysis showing E-cadherin, N-cadherin, Vimentin, SNAI1 and PMEPA1 protein levels in PMEPA1-OE HUCCT1 and RBE compared to NC. The column diagram showed the expression level of E-cadherin, N-cadherin, Vimentin, SNAI1 and PMEPA1 proteins. Data are presented as mean values with standard deviations. Statistical analysis used the two-sided t-test for comparisons between two groups and one-way ANOVA with Bonferroni correction for comparisons among multiple groups. For CCK-8 assays used two-way ANOVA with Bonferroni correction. (ns, not significant, **P*<0.05, ***P*<0.01, ****P*<0.001, n = 3)

**Supplementary Fig. S7 PMEPA1 regulates Hippo-YAP signaling to drive EMT in BTC.** (A, B) Western blotting analysis of the expression levels of Hippo pathway-related marker genes and PMEPA1 protein in RBE cells, and bar graphs showing the statistical analysis of expression levels. (C) Immunofluorescence staining images of YAP1 (red) showing its cellular localization in RBE cells. The nucleus is stained with DAPI (blue). The relative fluorescence intensity of YAP1 in the cytoplasm or nucleus was quantified and presented as a ratio to the total cellular fluorescence (Scale bar= 100 μm). (D, E) Western blotting analysis of YAP1 and p-YAP1 expression levels and corresponding statistical bar graph in RBE cells. Compared to the control group, nuclear expression was significantly reduced. GAPDH was used as a cytoplasm normalization control, while Lamin B1 was employed as a nucleus normalization control. (F) Western blotting analysis of the expression levels of Hippo pathway-related marker genes and PMEPA1 protein in RBE cells, and bar graphs showing the statistical analysis of expression levels. (G) Immunofluorescence staining images of YAP1 (red) showing its cellular localization in RBE cells. The nucleus is stained with DAPI (blue). The relative fluorescence intensity of YAP1 in the cytoplasm or nucleus was quantified and presented as a ratio to the total cellular fluorescence (Scale bar= 100 μm. (H) Western blotting analysis of YAP1 and p-YAP1 expression levels and corresponding statistical bar graph in RBE cells. Compared to the control group, nuclear expression was significantly reduced. GAPDH was used as a cytoplasm normalization control, while Lamin B1 was employed as a nucleus normalization control. (I) Western blotting analysis of the expression levels of EMT related marker genes and PMEPA1 protein in RBE cells, and bar graphs showing the statistical analysis of expression levels. Data are presented as mean values with standard deviations. Statistical analysis used the two-sided t-test for comparisons between two groups and one-way ANOVA with Bonferroni correction for comparisons among multiple groups. (ns, not significant, **P*<0.05, ***P*<0.01, ****P*<0.001, n = 3)

**Supplementary Fig. S8 Screening of EMT-targeted therapeutic drugs for BTC.** (A) Box plots showing oxaliplatin expression levels between the EMT (+) and EMT (−) groups based on the GDSC database. (B) UMAP plot showing the gemcitabine, Epoilone B, Docetaxel and SN-38-1494 and Vinorelbine scores for EMT (+) epithelial cells from the GDSC database. (C) Western blotting showing the comparison of E-cadherin, N-cadherin, Vimentin, SNAI1, YAP1 and PMEPA1 expression level in RBE after treatment with treated with seven different drugs. (D) Statistical analysis of the number of migrating and invading HUCCT1 cells. (E) Transwell assays revealing changes in RBE after treatment with SN-38 (Scale bar= 200 μm). Statistical analysis of the number of migrating and invading RBE cells. (F) Immunohistochemical detection of the expression level of YAP1 in the metastatic lung tissues of mice (Scale bar= 50 μm and 20 μm). (G) Western blotting showing the YAP1 and PMEPA1 expression level of PMEPA1 in the metastatic lung tissues of mice and bar graphs showing the statistical analysis of expression levels. Data are presented as mean values with standard deviations. Statistical analysis used one-way ANOVA with Bonferroni correction for comparisons among multiple groups. The fluorescence imaging of mic used two-way ANOVA with Bonferroni correction. The number of nude mouse tail vein lung metastasis model samples was 5. (ns, not significant, **P*<0.05, ***P*<0.01, ****P*<0.001, n = 3)

**Supplementary Table S1. scRNA-seq sample information**

| **Sample ID** | **Orig.ident** | **Tissue type** | **Data source** |
| --- | --- | --- | --- |
| N01 | GSM5237039_Sample_01_healthy_ | iCCA | GSM5237039 |
| N02 | GSM5237040_Sample_02_healthy_ | iCCA | GSM5237040 |
| N03 | GSM5237041_Sample_03_healthy_ | iCCA | GSM5237041 |
| N04 | GSM5237042_Sample_04_healthy_ | iCCA | GSM5237042 |
| N05 | GSM5237043_Sample_05_healthy_ | iCCA | GSM52370403 |
| N06 | GSM5237044_Sample_06_healthy_ | iCCA | GSM5237044 |
| N07 | X18P | iCCA | GSM4116579 |
| N08 | X23P | iCCA | GSM4116582 |
| N09 | X25P | iCCA | GSM4116586 |
| N10 | 3CN | iCCA | GSM5709332 |
| N11 | GSE181878_P | iCCA | GSM5513590 |
| N12 | P1N | pCCA | Original sequencing data |
| N13 | P2N | pCCA | Original sequencing data |
| N14 | P3N | pCCA | Original sequencing data |
| T01 | GSM5237045_Sample_01_tumor_ | iCCA | GSM5237045 |
| T02 | GSM5237046_Sample_02_tumor_ | iCCA | GSM5237046 |
| T03 | GSM5237047_Sample_03_tumor_ | iCCA | GSM5237047 |
| T04 | GSM5237048_Sample_04_tumor_ | iCCA | GSM5237048 |
| T05 | GSM5237049_Sample_05_tumor_ | iCCA | GSM5237049 |
| T06 | GSM5237050_Sample_06_tumor_ | iCCA | GSM5237050 |
| T07 | GSM6063506_230093_primary | iCCA | GSM6063506 |
| T08 | GSM6063510_230093_metastasis | iCCA | GSM6063510 |
| T09 | GSM6063512_230498_primary | dCCA | GSM6063512 |
| T10 | GSM6063514_230498_lymph_node | dCCA | GSM6063514 |
| T11 | GSM6063518_357818_primary | iCCA | GSM6063518 |
| T12 | GSM6063522_357818_metastasis | iCCA | GSM6063522 |
| T13 | GSM6063524_230723_primary_focus | GBC | GSM6063524 |
| T14 | GSM6063526_230723_lymph_node | GBC | GSM6063526 |
| T15 | GSM6063528_230723_metastasis | GBC | GSM6063528 |
| T16 | GSM6063532_231260_primary | GBC | GSM6063532 |
| T17 | GSM6063534_231260_lymph | GBC | GSM6063534 |
| T18 | GSM6416064 | eCCA | GSM6416064 |
| T19 | GSM6416065 | iCCA | GSM6416065 |
| T20 | X18T | iCCA | GSM4116580 |
| T21 | X20T | iCCA | GSM4116581 |
| T22 | X23T | iCCA | GSM4116583 |
| T23 | X24T1 | iCCA | GSM4116584 |
| T24 | X24T2 | iCCA | GSM4116585 |
| T25 | C60 | iCCA | GSM4050106 |
| T26 | C66 | iCCA | GSM4050110 |
| T27 | 2CT1 | iCCA | GSM5709309 |
| T28 | 2CT2 | iCCA | GSM5709310 |
| T29 | 3CT2 | iCCA | GSM5709311 |
| T30 | 2CB | iCCA | GSM5709331 |
| T31 | P1T | pCCA | Original sequencing data |
| T32 | P2T | pCCA | Original sequencing data |
| T33 | P3T | pCCA | Original sequencing data |

**Supplementary Table S2. RNA-seq sample information**

| **GSE number** | **GSM number** | | | **Cell/Tissue type** | **Team** | **Data typing** |
| --- | --- | --- | --- | --- | --- | --- |
| GSE107943 | GSM2883928 | | | Intrahepatic cholangiocarcioma | Tumor | RNA-seq |
| GSE107943 | GSM2883929 | | | Intrahepatic cholangiocarcioma | Tumor | RNA-seq |
| GSE107943 | GSM2883930 | | | Intrahepatic cholangiocarcioma | Tumor | RNA-seq |
| GSE107943 | GSM2883931 | | | Intrahepatic cholangiocarcioma | Tumor | RNA-seq |
| GSE107943 | GSM2883932 | | | Intrahepatic cholangiocarcioma | Tumor | RNA-seq |
| GSE107943 | GSM2883933 | | | Intrahepatic cholangiocarcioma | Tumor | RNA-seq |
| GSE107943 | GSM2883934 | | | Intrahepatic cholangiocarcioma | Tumor | RNA-seq |
| GSE107943 | GSM2883935 | | | Intrahepatic cholangiocarcioma | Tumor | RNA-seq |
| GSE107943 | GSM2883936 | | | Intrahepatic cholangiocarcioma | Tumor | RNA-seq |
| GSE107943 | GSM2883937 | | | Intrahepatic cholangiocarcioma | Tumor | RNA-seq |
| GSE107943 | GSM2883938 | | | Intrahepatic cholangiocarcioma | Tumor | RNA-seq |
| GSE107943 | GSM2883939 | | | Intrahepatic cholangiocarcioma | Tumor | RNA-seq |
| GSE107943 | GSM2883940 | | | Intrahepatic cholangiocarcioma | Tumor | RNA-seq |
| GSE107943 | GSM2883941 | | | Intrahepatic cholangiocarcioma | Tumor | RNA-seq |
| GSE107943 | GSM2883942 | | | Intrahepatic cholangiocarcioma | Tumor | RNA-seq |
| GSE107943 | GSM2883943 | | | Intrahepatic cholangiocarcioma | Tumor | RNA-seq |
| GSE107943 | GSM2883944 | | | Intrahepatic cholangiocarcioma | Tumor | RNA-seq |
| GSE107943 | GSM2883945 | | | Intrahepatic cholangiocarcioma | Tumor | RNA-seq |
| GSE107943 | GSM2883946 | | | Intrahepatic cholangiocarcioma | Tumor | RNA-seq |
| GSE107943 | GSM2883947 | | | Intrahepatic cholangiocarcioma | Tumor | RNA-seq |
| GSE107943 | GSM2883948 | | | Intrahepatic cholangiocarcioma | Tumor | RNA-seq |
| GSE107943 | GSM2883949 | | | Intrahepatic cholangiocarcioma | Tumor | RNA-seq |
| GSE107943 | GSM2883950 | | | Intrahepatic cholangiocarcioma | Tumor | RNA-seq |
| GSE107943 | GSM2883951 | | | Intrahepatic cholangiocarcioma | Tumor | RNA-seq |
| GSE107943 | GSM2883952 | | | Intrahepatic cholangiocarcioma | Tumor | RNA-seq |
| GSE107943 | GSM2883953 | | | Intrahepatic cholangiocarcioma | Tumor | RNA-seq |
| GSE107943 | GSM2883954 | | | Intrahepatic cholangiocarcioma | Tumor | RNA-seq |
| GSE107943 | GSM2883955 | | | Intrahepatic cholangiocarcioma | Tumor | RNA-seq |
| GSE107943 | GSM2883956 | | | Intrahepatic cholangiocarcioma | Tumor | RNA-seq |
| GSE107943 | GSM2883957 | | | Intrahepatic cholangiocarcioma | Tumor | RNA-seq |
| GSE107943 | GSM2883958 | | | Intrahepatic cholangiocarcioma | Normal | RNA-seq |
| GSE107943 | GSM2883959 | | | Intrahepatic cholangiocarcioma | Normal | RNA-seq |
| GSE107943 | GSM2883960 | | | Intrahepatic cholangiocarcioma | Normal | RNA-seq |
| GSE107943 | GSM2883961 | | | Intrahepatic cholangiocarcioma | Normal | RNA-seq |
| GSE107943 | GSM2883962 | | | Intrahepatic cholangiocarcioma | Normal | RNA-seq |
| GSE107943 | GSM2883963 | | | Intrahepatic cholangiocarcioma | Normal | RNA-seq |
| GSE107943 | GSM2883964 | | | Intrahepatic cholangiocarcioma | Normal | RNA-seq |
| GSE107943 | GSM2883965 | | | Intrahepatic cholangiocarcioma | Normal | RNA-seq |
| GSE107943 | GSM2883966 | | | Intrahepatic cholangiocarcioma | Normal | RNA-seq |
| GSE107943 | GSM2883967 | | | Intrahepatic cholangiocarcioma | Normal | RNA-seq |
| GSE107943 | GSM2883968 | | | Intrahepatic cholangiocarcioma | Normal | RNA-seq |
| GSE107943 | GSM2883969 | | | Intrahepatic cholangiocarcioma | Normal | RNA-seq |
| GSE107943 | GSM2883970 | | | Intrahepatic cholangiocarcioma | Normal | RNA-seq |
| GSE107943 | GSM2883971 | | | Intrahepatic cholangiocarcioma | Normal | RNA-seq |
| GSE107943 | GSM2883972 | | | Intrahepatic cholangiocarcioma | Normal | RNA-seq |
| GSE107943 | GSM2883973 | | | Intrahepatic cholangiocarcioma | Normal | RNA-seq |
| GSE107943 | GSM2883974 | | | Intrahepatic cholangiocarcioma | Normal | RNA-seq |
| GSE107943 | GSM2883975 | | | Intrahepatic cholangiocarcioma | Normal | RNA-seq |
| GSE107943 | GSM2883976 | | | Intrahepatic cholangiocarcioma | Normal | RNA-seq |
| GSE107943 | GSM2883977 | | | Intrahepatic cholangiocarcioma | Normal | RNA-seq |
| GSE107943 | GSM2883978 | | | Intrahepatic cholangiocarcioma | Normal | RNA-seq |
| GSE107943 | GSM2883979 | | | Intrahepatic cholangiocarcioma | Normal | RNA-seq |
| GSE107943 | GSM2883980 | | | Intrahepatic cholangiocarcioma | Normal | RNA-seq |
| GSE107943 | GSM2883981 | | | Intrahepatic cholangiocarcioma | Normal | RNA-seq |
| GSE107943 | GSM2883982 | | | Intrahepatic cholangiocarcioma | Normal | RNA-seq |
| GSE107943 | GSM2883983 | | | Intrahepatic cholangiocarcioma | Normal | RNA-seq |
| GSE107943 | GSM2883984 | | | Intrahepatic cholangiocarcioma | Normal | RNA-seq |
| **Supplementary Table S3. BTC EMT key gene coming from NMF** | | | | | | |
|  | | | **Gene name** | | | |
| BTC EMT core gene list | | | COL6A2, PVR, GJA1, APLP1, PDLIM4, FERMT2, COL4A1, LOXL2, AREG, JUN, TIMP1, TPM4, SERPINH1, MGP, SPARC, TPM2, COL1A2, CALD1, EFEMP2, FN1, LAMC2, CXCL8, TNFRSF12A, IL32, CXCL1, PLAUR, IGFBP3, VIM, TPM1, LGALS1, TAGLN, MYL9, MMP1, SPP1, PRSS2, CXCL6, CTHRC1, FSTL3, DKK1, PMEPA1, ITGA2, SERPINE1, FLNA, CAPG, EMP3, CD59, ITGB1, TGM2, SPOCK1, SERPINE2, HTRA1. | | | |
| **Supplementary Table S4. Comment gene from multiple data sets** | | | | | | |
|  | | **Gene name** | | | | |
| 198 up_regulated | | A4GALT, ABHD17C, ADAM9, AGRN, AHNAK2, ALDOA, ANO9, ANXA2, ARHGEF16, ATP1B3, B3GNT3, BACE2, BAIAP2L2, BIK, C15orf48, C16orf74, C19orf33, C4orf48, CAPG, CAPN8, CAPS, CD44, CD58, CDCP1, CDH3, CDKN2A, CEACAM6, CKB, CLDN18, CLDN4, CLDN7, CLIC3, COL17A1, CRABP2, CRNDE, CRYAB, CTHRC1, CTSC, CTSE, CXCL5, CYBA, DBNDD2, DDR1, DKK1, DMKN, DSG2, DUOX2, DUOXA2, EGLN3, ELMO3, ENO2, EPS8L1, EPS8L3, ESRP1, EVPL, FERMT1, FKBP10, FLNA, FOLR1, FUT3, FXYD3, G6PD, GCNT3, GMNN, GOLM1, GPRC5A, GRB7, GSTP1, HES4, HIST3H2A, HK2, HMGA1, HOXB7, IL18, ISYNA1, ITGA2, ITGA3, ITGB4, ITGB6, ITIH5, ITPR3, KCNN4, KLF5, KLK10, KLK11, KRT15, KRT17, KRT19, KRT23, KRT7, KRTCAP3, LAMA3, LAMA5, LAMB3, LAMC2, LCN2, LGALS3, LINC00152, LIPH, LMTK3, LPCAT4, LYPD6B, MALL, MAPK13, MBOAT2, MDFI, MDK, MFSD10, MIR4435-1HG, MISP, MMP1, MMP28, MSLN, MST1R, MTHFD2, MUC1, MUC13, MUC4, MYEOV, MYOF, NMB, NQO1, PAFAH1B3, PCSK1N, PDGFA, PDLIM4, PDLIM7, PDZK1IP1, PFKP, PGM2L1, PHLDA3, PI3, PKM, PKP3, PLAU, PLP2, PMAIP1, PMEPA1, PROM2, PRSS22, PSORS1C1, PTGES, PTHLH, PTTG1, PYCARD, PYCR1, PYGB, QSOX1, RAB11FIP1, RAB25, RAB34, RASSF6, RHOF, RHOV, RUSC1, S100A1, S100A11, S100A14, S100A2, S100A4, S100A6, SCNN1A, SERPINE2, SEZ6L2, SFN, SFTA2, SLC16A3, SLC1A5, SLC2A1, SLC44A2, SLC44A4, SLC6A8, SMIM22, SPINK1, SPINT1, SPINT2, ST14, SYT8, SYTL1, TFAP2A, TFF1, TM4SF1, TMC5, TMC6, TMED3, TMEM159, TMEM54, TMPRSS4, TMSB10, TNNT1, TPM2, TPM4, TRIM31, TSPAN15, UBE2C, UCA1, UCHL1, WFDC2 | | | | |
| 351 down_regulated | | A1BG, A1CF, A2M, AADAC, ABAT, AC004862.6, ACAA1, ACAA2, ACADM, ACADS, ACADSB, ACAT1, ACAT2, ACMSD, ACOT2, ACOX2, ACSL1, ACSM2A, ACSM2B, ACSM3, ACSM5, ACY1, ADH1A, ADH1B, ADH4, ADH6, ADI1, AFM, AGMAT, AGXT, AHSG, AK3, AKR1C4, AKR1D1, AKR7A3, ALAD, ALAS1, ALB, ALDH1A1, ALDH1B1, ALDH1L1, ALDH2, ALDH4A1, ALDH6A1, ALDH7A1, ALDH8A1, ALDOB, AMBP, AMDHD1, AMT, ANG, ANGPTL3, ANXA10, AOX1, APCS, APOA1, APOA2, APOB, APOC1, APOC2, APOC3, APOE, APOH, APOM, ARG1, ASGR1, ASL, ASPDH, ASPG, ASS1, ATF5, AZGP1, BAAT, BBOX1, BCHE, BCKDHB, BDH1, BDH2, BHMT, BHMT2, BPHL, C4BPA, C4BPB, C6, C7orf55, C8B, C9, CAT, CBR1, CBS, CD14, CD302, CDO1, CEBPA, CES1, CFHR1, CFHR3, CFI, CFL2, CLU, CLYBL, CMBL, CP, CPB2, CPT2, CREB3L3, CRYL1, CSAD, CTH, CXCL2, CYB5A, CYP27A1, CYP2C8, CYP2C9, CYP2D6, CYP2E1, CYP3A4, CYP4A11, DAK, DAO, DCXR, DDT, DECR1, DHODH, DHRS1, DHRS4L2, DHTKD1, DNASE1L3, DPYS, DUSP1, ECHDC2, ECHDC3, ECHS1, ECI2, EHHADH, EPB41L4B, EPHX1, EPHX2, ESPN, ETFDH, ETNK2, ETNPPL, F2, FABP1, FAH, FAM13A, FAM46A, FBP1, FCGRT, FCN3, FERMT2, FGA, FGB, FGG, FGGY, FGL1, FMO3, FMO5, FOS, FTCD, FTL, FUOM, FXN, FXYD1, G6PC, GADD45B, GADD45G, GALK1, GALT, GAMT, GATM, GC, GCDH, GCSH, GK, GLRX, GLUD1, GLYATL1, GLYCTK, GNMT, GOT1, GPD1, GPT, GPT2, GRHPR, GSTA1, GSTZ1, HAAO, HADH, HAGH, HAMP, HAO1, HAO2, HBB, HFE2, HGD, HIGD1A, HMGCL, HMGCS2, HNF4A, HP, HPD, HPGD, HPR, HPX, HRG, HRSP12, HSD11B1, HSD17B13, HSD17B4, HSD17B6, HSD17B8, HULC, ID2, IDNK, IGFBP1, IQGAP2, ISOC1, ITIH1, ITIH2, KDM8, KHK, KNG1, LBP, LCAT, LDHD, LEAP2, LINC00261, LRG1, LYRM5, MASP2, MAT1A, METTL7A, MGMT, MGST1, MLXIPL, MMAB, MPC1, MSRA, MT1A, MT1E, MT1F, MT1G, MT1H, MT1M, MT1X, MT2A, MTHFD1, MTHFS, MUT, N4BP2L1, NADK2, NDRG2, NNMT, NR0B2, NR1H4, NR1I3, NUDT7, ORM1, ORM2, OTC, PAH, PALMD, PANK1, PBLD, PCCB, PCK1, PCK2, PEBP1, PECR, PEPD, PHGDH, PHYH, PHYHD1, PIPOX, PLG, PLGLB1, PLIN2, PON1, PON3, PPP1R1A, PRAP1, PRDX3, PROC, PRODH2, PSAT1, PTGR1, PXMP2, QDPR, QPRT, RARRES2, RBP4, RBP5, RCAN1, RCL1, RDH16, RGN, RHOB, RNASE4, RP11-119D9.1, SAA4, SAT2, SCP2, SDC2, SDHB, SDS, SEC14L2, SELENBP1, SEPP1, SERPINA1, SERPINA11, SERPINA4, SERPINA5, SERPINA6, SERPINC1, SERPIND1, SERPING1, SHMT1, SLC22A1, SLC25A47, SLC2A2, SLC9A3R2, SMIM14, SMLR1, SOD1, SORD, SPRYD4, SUCLG2, SULT1A1, SULT1A2, SULT2A1, TAT, TCEA3, TDO2, TF, TMEM176A, TMEM176B, TMEM220, TMEM56, TTC36, TTC38, TTC39C, TTPA, TTR, UGP2, UGT2B15, UGT2B4, UGT2B7, UPB1, VNN1, VTN | | | | |

| **Supplementary Table S5. Reagent for experiment** | | | |
| --- | --- | --- | --- |
| **Reagent name** | **Manufacturers** | **Item number** | **Experiment** |
| PMEPA1 Monoclonal antibody | Proteintech | 16521-1-AP | Western blotting |
| E-cadherin Monoclonal antibody | Proteintech | 60335-1-Ig | Western blotting |
| N-cadherin Polyclonal antibody | Proteintech | 22018-1-AP | Western blotting |
| Vimentin Polyclonal antibody | Proteintech | 10336-1-Ap | Western blotting |
| SNAI1 Polyclonal antibody | Proteintech | 13099-1-AP | Western blotting |
| LATS1 Polyclonal antibody | Proteintech | 17049-1-AP | Western blotting |
| p-LATS1 Polyclonal antibody | Proteintech | 28998-1 | Western blotting |
| YAP1 Polyclonal antibody | ABclonal | A21216 | Western blotting |
| p-YAP1 Polyclonal antibody | Diagbio | db13334 | Western blotting |
| CYR61 Polyclonal antibody | Proteintech | 26689-1-AP | Western blotting |
| UltraSYBR Mixture | CWBIO | CW0957 | RT-qPCR |
| Ultrapure RNA Kit | CWBIO | CW0581 | RT-qPCR |
| HiFiScript gDNA Removal cDNA Synthesis Kit | CWBIO | CW2582 | RT-qPCR |
| Protease Inhibitor Cocktail | MedChemExpress | [HY-K0010](https://www.medchemexpress.cn/inhibitor-kit/protease-inhibitor-cocktail.html) | Western blotting |
| SDS-PAGE loading buffer | Solarbio | P1016 | Western blotting |
| PVDF membrane | Millipore | ISEQ00010 | Western blotting |
| ECL chemiluminescence kit | Proteintech | PK10003 | Western blotting |
| Normal goat serum | CWBIO | CW0130S | Immunohistochemistry |
| HRP-conjugated secondary antibody | Proteintech | PK10006 | Immunohistochemistry |
| DAB | Beyotime | P0202 | Immunohistochemistry |
| CoraLite488-conjugated Goat Anti-Rabbit IgG(H+L) | Proteintech | SA00013-2 | Immunofluorescence |
| CoraLite594 – conjugated Goat Anti-Mouse IgG(H+L) | Proteintech | SA00013-3 | Immunofluorescence |
| Mounting Medium With DAPI | Abcam | ab104139 | Immunofluorescence |
| Normal Goat Serum for Blocking | CWBIO | CW0130 | Immunofluorescence |

**Supplementary Table S6. Target Sequences for stable cell lines**

| **Name** | **Sequences** **(5' to 3')** | **Manufacturers** |
| --- | --- | --- |
| shNC | TTCTCCGAACGTGTCACGT | Gene Chem |
| shPMEPA1#1 | CTGGAGCAAAGAGAAGGATAA | Gene Chem |
| shPMEPA1#2 | CATCTTCGACAGTGACCTGAT | Gene Chem |
| PMEPA1-OE | TTTCCGGTGAATTCCTCGAGCGCCACC | Gene Chem |

**Supplementary Table S7. Sequences for Primer**

| **Primer Name** | **Sequences** **(5' to 3')** |
| --- | --- |
| ACTB-F | ATCAAGATCATTGCTCCTCCTG |
| ACTB- R | GACTCCCGGTTCAGTTCCAG |
| PMEPA1-a-F | CTGCAAACGCTCTTTGTTCCA |
| PMEPA1-a-R | TGCAGACAGCTTGTAGTGGC |
| PMEPA1-b-F | ACGCGAGTTCCCGTCTTTC |
| PMEPA1-b-R | AAACAAACTCCAGCTCCGCCA |
| PMEPA1-c-F | GCGAGTTCCCGTCTTTCCTG |
| PMEPA1-c-R | TGAACAAACTCCAGCTCCGCTG |
| PMEPA1-d-F | GACCCTCTACACCCGCCATA |
| PMEPA1-d-R | GGCATTTTGACTTTTCGCCTG |
| PMEPA1-e-F | CTTCCCCGTGTGCAAGAG |

**Supplementary Table S8. Drugs for experiment**

| Drug | Concentration (μM) | Catalog Number | Supplier |
| --- | --- | --- | --- |
| TRULI | 10 | HY-138489 | MedChemExpress |
| Docetaxel | 0.05 | HY-B0011 | MedChemExpress |
| Epothilone B | 1 | HY-17029 | MedChemExpress |
| Elesclomol | 0.5 | HY-12040 | MedChemExpress |
| Gemcitabine | 0.2 | HY-17026 | MedChemExpress |
| Oxaliplatin | 50 | HY-17371 | MedChemExpress |
| SN-38 | 1 | HY-13704 | MedChemExpress |
| Vinorelbine | 0.06 | HY-12053A | MedChemExpress |
