# Supplementary material for: PMEPA1 modulates YAP1 nuclear translocation to disrupt EMT subtypes and promote metastasis in Biliary tract cancer
Source: Cell Death Dis. 2026 Apr 3;17(1):449. doi: 10.1038/s41419-026-08684-3 (PMC13172513; doi:10.1038/s41419-026-08684-3)

Unedited blot and gel images

Fig 6B

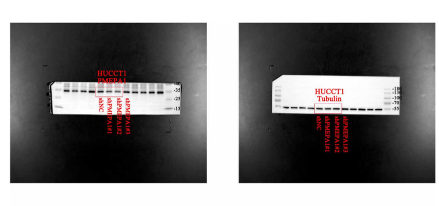

Fig S6B

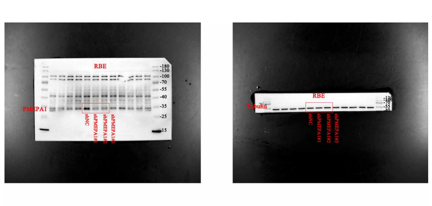

Fig 6J

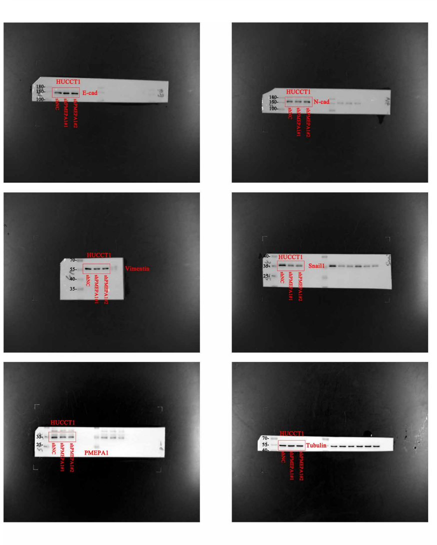

Fig S6H

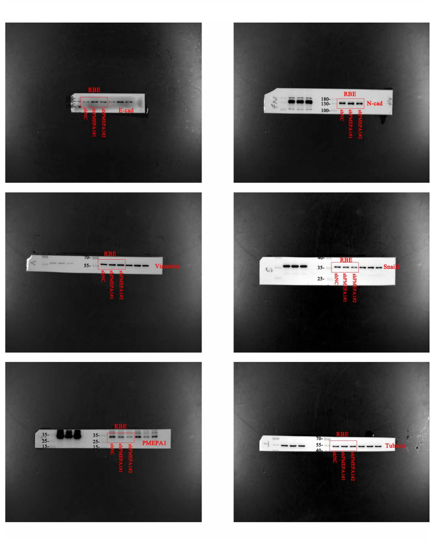

FIG S6K

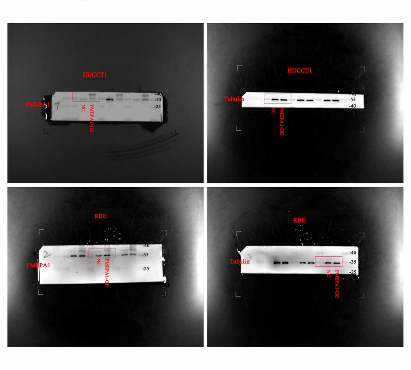

FIG S6O HUCCT1

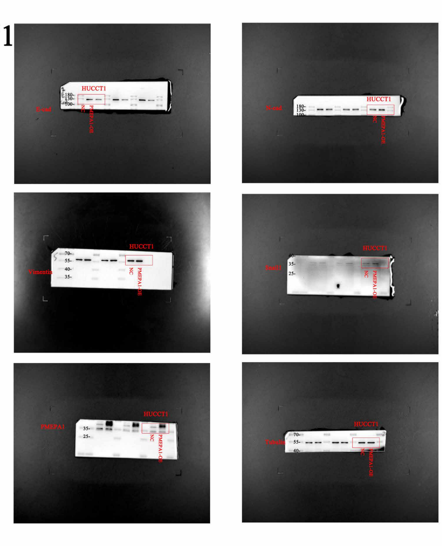

FIG S6O RBE

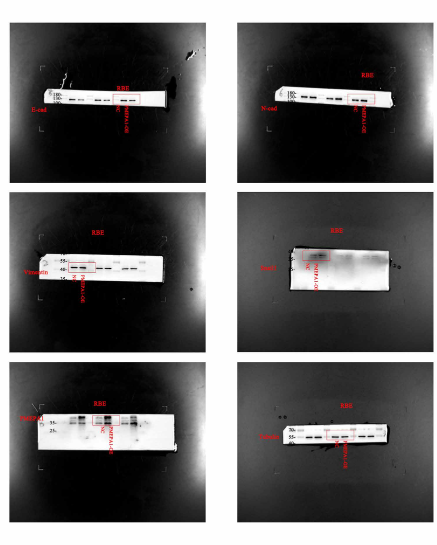

FIG 7A HUCCT1

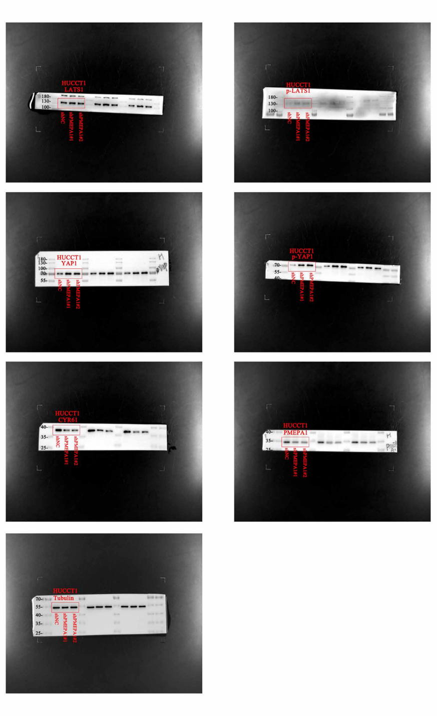

FIG S7A RBE

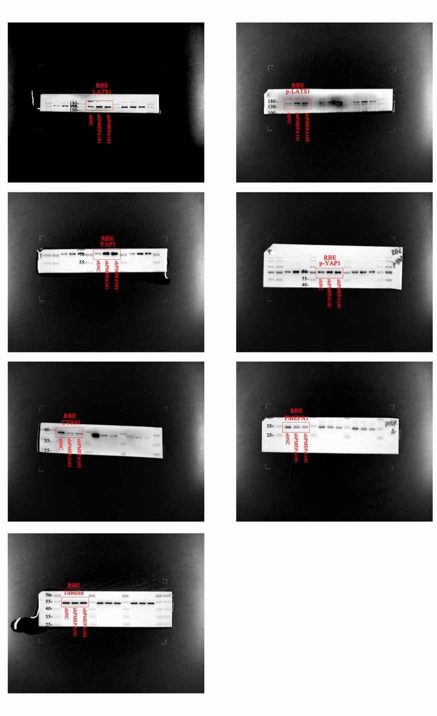

FIG 7A HUCCT1 OE

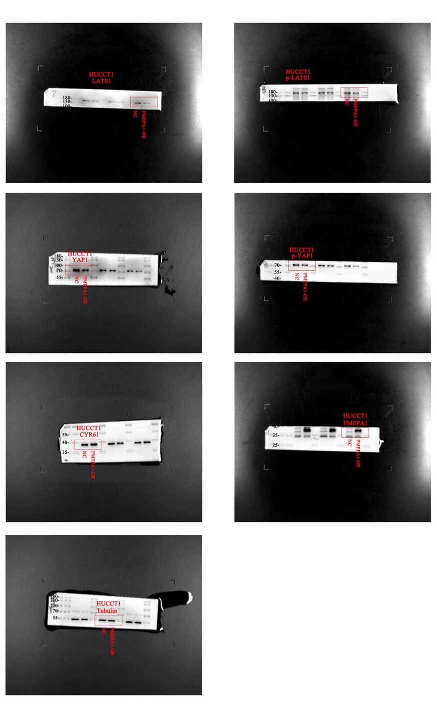

FIG 7A RBE OE

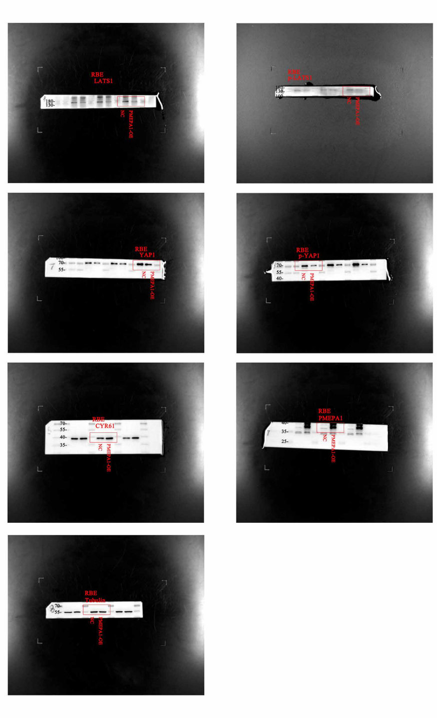

FIG 7D HUCCT1

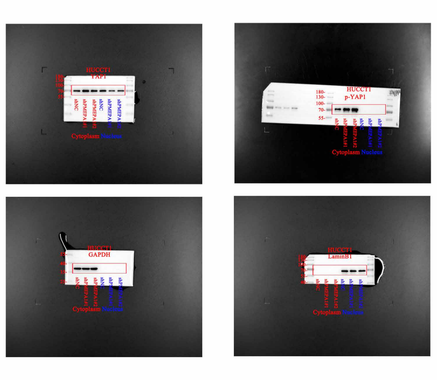

FIG S7D RBE

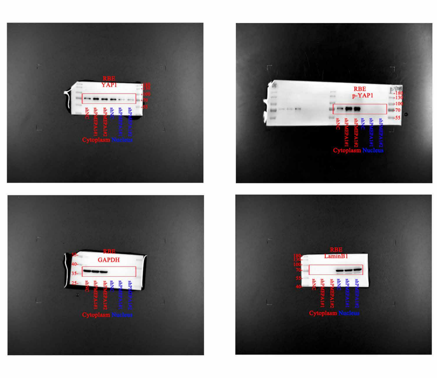

FIG 7F HUCCT1

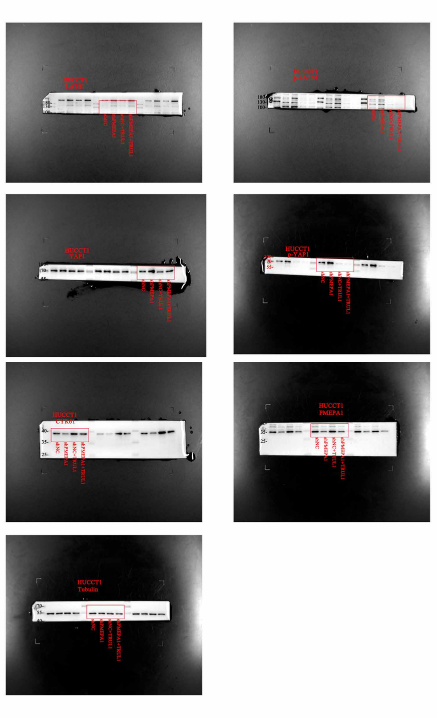

FIG S7F RBE

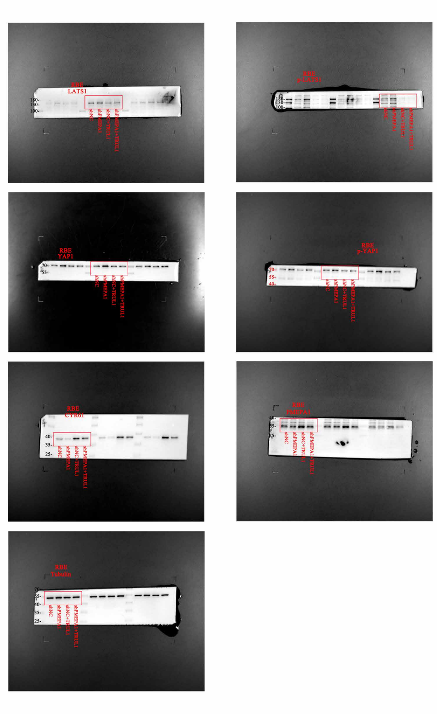

FIG 7H HUCCT1

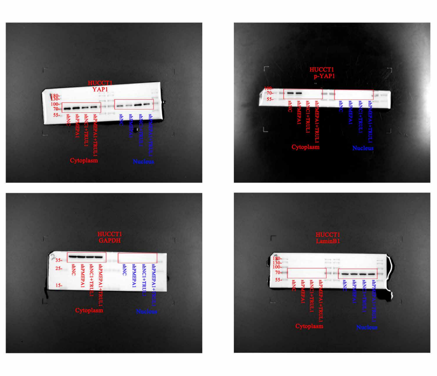

FIG S7H RBE

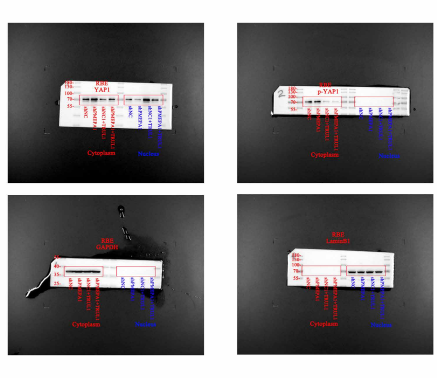

FIG 7I HUCCT1

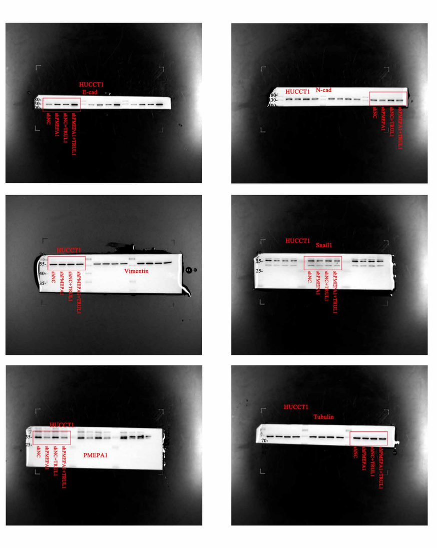

FIG S7I RBE

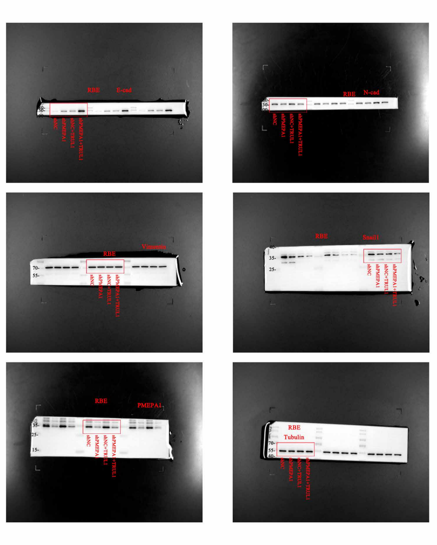

FIG 8C HUCCT1

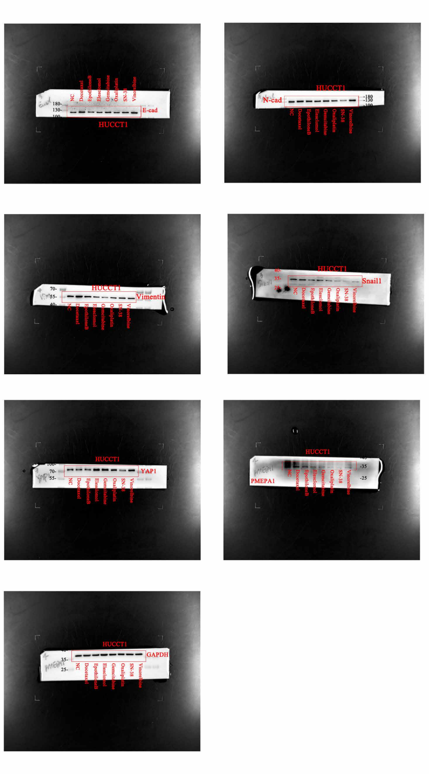

FIG S8C RBE

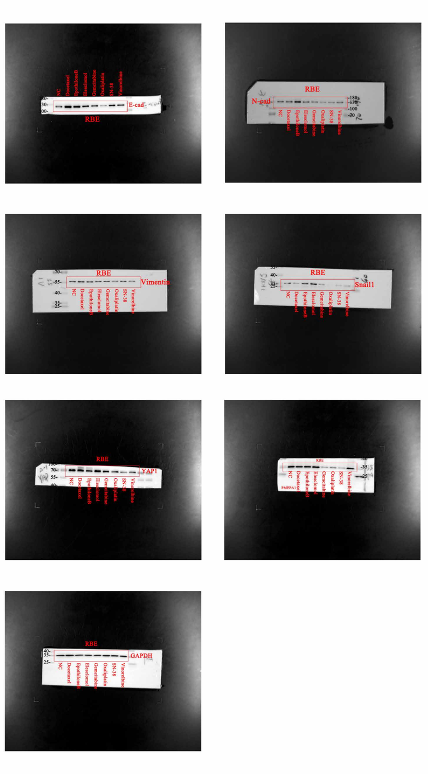

FIG S8G

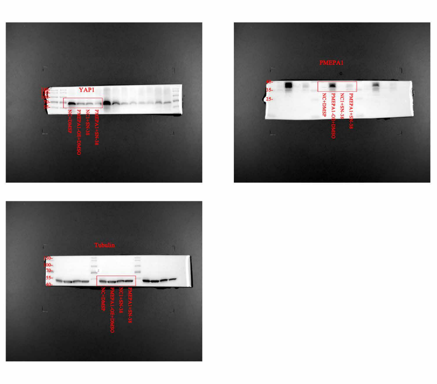

Supplement: Supplementary file 2 — Unedited blot and gel images [file 41419_2026_8684_MOESM2_ESM.pdf]
